# Phenome-wide Mendelian randomization analysis reveals multiple health comorbidities of coeliac disease

**DOI:** 10.1016/j.ebiom.2024.105033

**Published:** 2024-02-21

**Authors:** Shuai Yuan, Fangyuan Jiang, Jie Chen, Benjamin Lebwohl, Peter H.R. Green, Daniel Leffler, Susanna C. Larsson, Xue Li, Jonas F. Ludvigsson

**Affiliations:** a School of Public Health and the Second Affiliated Hospital, Zhejiang University School of Medicine, Hangzhou, Zhejiang, China; b Unit of Cardiovascular and Nutritional Epidemiology, Institute of Environmental Medicine, Karolinska Institutet, Stockholm, Sweden; c Department of Medicine, Celiac Disease Center at Columbia University Medical Center, New York, NY, USA; d Departments of Medicine and Surgical Pathology, Columbia University College of Physicians and Surgeons, New York, NY, USA; e The Celiac Center at Beth Israel Deaconess Medical Center, Harvard Medical School, USA; f Unit of Medical Epidemiology, Department of Surgical Sciences, Uppsala University, Uppsala, Sweden; g Department of Medical Epidemiology and Biostatistics, Karolinska Institutet, Stockholm, Sweden; h Department of Pediatrics, Orebro University Hospital, Orebro, Sweden

**Keywords:** Celiac disease, Coeliac disease, Comorbidities, Health effect, Mendelian randomization

## Abstract

**Background:**

Coeliac disease (CeD) has been associated with a broad range of diseases in observational data; however, whether these associations are causal remains undetermined. We conducted a phenome-wide Mendelian randomization analysis (MR-PheWAS) to investigate the comorbidities of CeD.

**Methods:**

Single nucleotide polymorphisms (SNPs) associated with CeD at the genome-wide significance threshold and without linkage disequilibrium (*R*^*2*^ <0.001) were selected from a genome-wide association study including 12,041 CeD cases as the instrumental variables. We first constructed a polygenic risk score for CeD and estimated its associations with 1060 unique clinical outcomes in the UK Biobank study (N = 385,917). We then used two-sample MR analysis to replicate the identified associations using data from the FinnGen study (N = 377,277). We performed a secondary analysis using a genetic instrument without extended *MHC* gene SNPs.

**Findings:**

Genetic liability to CeD was associated with 68 clinical outcomes in the UK Biobank, and 38 of the associations were replicated in the FinnGen study. Genetic liability to CeD was associated with a higher risk of several autoimmune diseases (type 1 diabetes and its complications, Graves' disease, Sjögren syndrome, chronic hepatitis, systemic and cutaneous lupus erythematosus, and sarcoidosis), non-Hodgkin's lymphoma, and osteoporosis and a lower risk of prostate diseases. The associations for type 1 diabetes and non-Hodgkin's lymphoma attenuated when excluding SNPs in the *MHC* region, indicating shared genetic aetiology.

**Interpretation:**

This study uncovers multiple clinical outcomes associated with genetic liability to CeD, which suggests the necessity of comorbidity monitoring among this population.

**Funding:**

This project was funded by 10.13039/501100004047Karolinska Institutet and the 10.13039/501100004359Swedish Research Council.

**Research in context**

**Evidence before this study**

Coeliac disease (CeD) is an immune-mediated disease characterized by small intestinal villus atrophy and inflammation. It occurs in about 0.5–1% of the Western population and appears to be increasing in incidence in many regions. CeD has been associated with the risk of many other comorbidities, including autoimmune disease, liver disorders, certain cancer, and malnutrition.

**Added value of this study**

This phenome-wide Mendelian randomization study explored the associations of genetic liability to CeD with a wide range of clinical outcomes in two large-scale biobanks. We first constructed a polygenic risk score to mimic the genetic predisposition to CeD and tested its association with 1060 clinical endpoints among 385,017 individuals in the UK Biobank. We found genetic liability to CeD associated with 68 clinical outcomes. These associations were consistent between women and men and different age strata. We then performed the two-sample Mendelian randomization analysis to confirm the identified associations using an external independent data source, the FinnGen study including 377,277 individuals. Thirty-eight associations were replicated. Taken together, genetic liability to CeD was associated with a higher risk of several autoimmune diseases, non-Hodgkin's lymphoma, osteoporosis, iron deficiency anaemias, and vitamin B-complex deficiencies and a lower risk of prostate diseases. In the analysis excluding genetic instruments in the *MHC* gene, the associations for type 1 diabetes and non-Hodgkin's lymphoma attenuated.

**Implications of all the available evidence**

This study found a wide range of clinical outcomes, in particular autoimmune diseases, non-Hodgkin's lymphoma, osteoporosis, and malnutrition, associated with CeD. *MHC* genotypes appeared to be dominantly important for the associations of CeD with T1D and non-Hodgkin's lymphoma. These findings reveal comorbidities of CeD and suggest the need for comorbidity monitoring in this population.

## Introduction

Coeliac disease (CeD) is an immune-mediated disease characterized by small intestinal villus atrophy and inflammation.^1^ It occurs in about 0.5–1% of the Western population and appears to be increasing in incidence in many regions.^2^ CeD has been linked to a large number of other disorders,^1^ and in many of these disorders testing for CeD is advised.^3^ For example, the risk of autoimmune disease,^4^ liver disorders,^5^ certain cancer,^6^ and malnutrition^7^ has been found to be increased among patients with CeD in population-based case–control or cohort studies. However, whether these links are causal or based on shared environmental risk factors remains largely unestablished due to potential drawbacks of observational studies, such as residual confounding, reverse causation, and misclassification. A clear appraisal of the causality of these associations can better inform CeD monitoring and screening.

CeD is triggered by gluten exposure, with tissue transglutaminase being the autoantigen.^8^^,^^9^ While environmental risk factors are clearly important, it has long been known that CeD has a genetic component,^10^, ^11^, ^12^ with a strong link to *HLA* (also known as the Major Histocompatibility complex [MHC])^13^ gene complex. Aside from CeD, the *MHC* gene complex exerts a broad influence on human health via immune response, regulation, and surveillance.^14^ Thus, whether the associations between CeD and other diseases heavily rely on this gene becomes interesting for deciphering the underlying mechanisms.

Mendelian randomization (MR) analysis is an epidemiological approach that can reinforce causal inference by using genetic variants as an instrumental variable to mimic the effect of the exposure.^15^ The method has two major merits including 1) minimizing confounding since genetic variants are randomly assorted at conception and therefore not associated with confounders (usually environmental and self-adopted factors), and 2) diminishing reverse causality because germline phenotype cannot be modified by the onset or progression of disease. An MR-Phenome-wide association study (MR-PheWAS) is an efficient way to examine the causality between the exposure and a wide range of clinical outcomes in a large-scale biobank.^16^ Here, we conducted an MR-PheWAS to explore the associations between genetic predisposition to CeD and a large number of diseases with the aim of pinpointing health comorbidities of CeD.

## Methods

### Study design and ethic permit

**Fig. 1:**
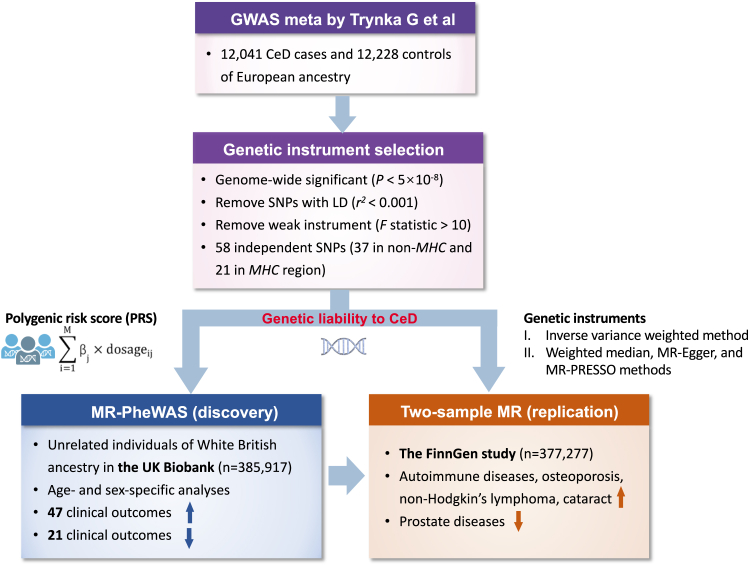
Schematic representation of the study design. CeD, coeliac disease; MR, Mendelian randomization; GWAS, genome-wide association study; LD, linkage disequilibrium; MR-PheWAS, phenome-wide Mendelian randomization analysis; SNPs, single nucleotide polymorphisms.

Fig. 1 shows the study design overview. We first conducted an MR-PheWAS to explore clinical outcomes associated with genetic liability to CeD in the UK Biobank study. To confirm the identified associations, we used the two-sample MR analysis in the FinnGen study as the replication. A secondary analysis using non-*MHC* genetic instruments was performed to examine whether these associations were driven by *MHC*. There are three assumptions of MR: 1) the genetic variants used as the instrumental variable should be robust associated with the exposure (i.e., CeD); 2) the genetic instruments should not be associate with any confounders; and 3) the genetic variants should influence the outcome only through exposure instead of through other alterative pathways.^15^

### Genetic instrument selection

Single nucleotide polymorphisms (SNPs) associated with CeD at the genome-wide significance level (*P* < 5 × 10^−8^) were selected from a genome-wide meta-analysis including 12,041 individuals with CeD (cases) and 12,228 controls of European ancestry (not including UK Biobank or FinnGen).^12^ CeD cases were identified based on established clinical criteria, corroborative serological findings, and, universally, through small intestinal biopsy.^12^ We estimated linkage disequilibrium (LD, i.e., genetic correlation matrix) among these SNPs using the 1000 Genomes European reference panel and removed SNPs with high LD (*R*^*2*^ >0.001). We calculated the *F* statistic (β^2^/standard error^2^) as the indicator of the strength of genetic instruments and found all *F* statistics >10 (average *F* statistic = 233.3), which indicates that weak instrument bias is less likely.^17^ A total of 58 independent SNPs strongly associated with CeD was used to construct a weighted polygenic risk score (PRS) in the MR-PheWAS and used as the instrumental variables in the two-sample MR analyses to proxy genetic liability to CeD (Supplementary Table S1). In the secondary analysis, we removed 21 SNPs in the *MHC* gene region that is defined between *HIST1H2AA* and *RPL12P1* regions (chromosome 6, GRCh37 position 25,726,291–33,368,421),^18^ leaving 37 SNPs as the instrumental variable. Assuming a population CeD prevalence of 1%, the used 58 and 37 SNPs explained approximately 45.3% and 6.5% of the genetic variance of CeD, respectively. We searched phenotypes associated with 37 SNPs not in *xMHC* in the PhenoScanner V2 database^19^ to explore whether used genetic instruments associated with pleiotropy. We found a few autoimmune-related traits associated with more than 5 (maximum 9) of 37 SNPs at the genome-wide significance threshold, which indicates limited pleiotropic effects.

### MR-PheWAS in the UK Biobank study

The UK Biobank is a large-scale ongoing population-based cohort study recruiting 500,000 participants aged 37–73 years from 2006 to 2010 across 22 assessment centres.^20^ The participants were invited to have a series of physical assessments and fill in questionnaires that elicited information on sociodemographic features, lifestyle factors, and self-reported health conditions. Biological samples were collected at the baseline for biochemical assays and genotyping. DNA sequencing was performed using the Affymetrix UK BiLEVE Axiom array and Affymetrix UK Biobank Axiom array (Affymetrix Research Services Laboratory, Santa Clara, CA, USA) and corresponding data were imputed using a reference panel combining the UK 10 K haplotype and the Haplotype Reference Consortium panels.^21^ To minimize population structure bias, the current analysis was restricted to 385,917 unrelated individuals of White British ancestry (Supplementary Figure S1). Clinical outcomes were defined by the PheCODE schema,^22^ which was based on 10,750 unique International Classification of Disease (ICD)-10 codes and 3113 ICD-9 codes with corresponding data from national medical records (inpatient hospital episode records, cancer registry, and death registry) in the UK Biobank. Detailed information on ICD code-PheCODE linking and quality control has been described in our previous studies.^23^^,^^24^

We calculated the Nagelkerke's *r*^*2*^ based on a subgroup (due to the imbalance between CeD and non-CeD numbers) of the UK Biobank participants to estimate the phenotypical variance explained by the constructed PRS. We first matched each patient with CeD to 5 non-CeD controls based on age and sex and then estimated the variance explained by the PRS on the observed scale using the Nagelkerke's *r*^*2*^, which was the difference in *r*^*2*^ between a logistic regression model including the PRS, sex, age, and top 10 principal components and a null logistic model comprising sex, age, and top 10 principal components.^25^ We found a Nagelkerke *r*^*2*^ of 24%, indicating a significant contribution of the PRS to the variability in celiac disease risk as well as the validity of used PRS as the proxy for CeD in the UK Biobank MR-PheWAS analysis.

### Two-sample MR in the FinnGen study

The FinnGen study is a growing study combining genotype data from Finnish biobanks and digital health record data from Finnish health registries.^26^ Participants were genotyped with Illumina and Affymetrix chip arrays (Illumina Inc., San Diego, and Thermo Fisher Scientific, Santa Clara, CA, USA), and chip genotype data were imputed using the population-specific SISu v4.0 imputation reference panel of 8554 whole genomes. Disease outcomes were defined using six national registries after harmonization of ICD-8, -9, and -10, cancer-specific ICD-O-3, (NOMESCO) procedure codes, Finnish-specific Social Insurance Institute (KELA) drug reimbursement codes, and ATC codes. Detailed information on quality control at sample- and gene-levels and association tests can be found in its webpage (https://finngen.gitbook.io/documentation/). The current analysis was based on the latest R9 data release.

### Statistical analysis

In the MR-PheWAS in the UK Biobank, we first constructed a weighted PRS of selected 58 SNPs identified in the GWAS meta-analysis by Trynka G et al.^12^ This PRS was designed to serve as a proxy for genetic liability to CeD. It comprehensively aggregates the effect of multiple genetic variants on CeD risk, quantifies the individualized genetic predisposition to CeD, and allows the exploration of sex- and age-specific associations in depth among the UK Biobank participants. The PRS was calculated using R Software 4.0.2 by summing up the number of CeD-increasing alleles for each SNP, each weighted by effect size on genetic susceptibility to CeD, and then adding this weighted score for all used SNPs. We removed clinical outcomes with a number of cases <120 to minimize the type 2 error rate.^23^ Subsequently, we estimated the associations between genetic liability to CeD quantified by the PRS and various clinical outcomes within the UK Biobank. These associations were assessed using logistic regression models with adjustment for age, sex, assessment centre, and the first ten principal components as indicators for population structure. Regrading sex-specific outcomes, the analysis was specifically performed in the corresponding sex. We performed a secondary analysis using 37 SNPs not in the *MHC* region to examine the effects of *MHC* on the identified associations. We also stratified the analysis by sex (women and men) and age (<60 and ≥ 60 years). The false discovery rate (FDR) correction with the method by Benjamini-Hochberg was employed to account for multiple comparisons in the MR-PheWAS analysis. The MR-PheWAS test was two-sided and implemented using a package by Carroll et al. in R Software 4.0.2.^27^

In the two-sample MR analysis in the FinnGen, the inverse variance weighted method under the multiplicative random effects was used as the primary analysis to estimate the associations between genetic liability to CeD and the risk of the identified clinical outcomes in the MR-PheWAS. Given that this analysis is sensitive to SNP outliers and subsequent horizontal pleiotropy, three sensitivity analyses, including the weighted median, MR-Egger, and MR-PRESSO methods, were performed to test the consistency of the results and detect and correct for possible horizontal pleiotropy. The weight median analysis can provide robust causal estimates assuming >50% weight from valid genetic instruments.^28^ The MR-Egger method can detect potential horizontal pleiotropy by its embedded intercept test (*P* < 0.05) and provide estimates after correcting for horizontal pleiotropy if any; however, the analysis is usually underpowered.^29^ The MR-PRESSO can detect outlying SNPs and generate estimates after the removal of the identified SNP outliers.^30^ The association with a *P* value < 0.05 was deemed significant and replicated. All tests were two-sided and performed by the TwoSampleMR package in R Software 4.0.2.^31^

To examine the second assumption of MR, we first pinpointed potential cofounders between CeD and related outcomes and then performed multivariable MR analysis with adjustment for genetically predicted confounders. There are a few risk factors identified for CeD, such as virus infection and dysbiosis of gut microbiota.^32^ Given no robust genetic instruments for virus infection (largely determined by virus exposure), we merely performed this analysis for gut microbiota. According to a compressively designed MR analysis on the association between gut microbiome species and CeD, genetically predicted abundance of *genus Bifidobacterium* was associated with CeD risk.^33^ Given no clear association between this microbiome specie and CeD-associated outcomes, we first examined the associations of genetically predicted abundance of *genus Bifidobacterium* with the risk of CeD-associated outcomes. In this analysis, we selected two genetic instruments (rs182549 and rs7322849) for *genus Bifidobacterium* at *P* < 5 × 10^−8^ and *R*^*2*^ <0.01 with data from the MiBioGen consortium including 18,340 individuals of European ancestry.^34^ Likewise, summary-level data for CeD-associated outcomes were obtained from the FinnGen R9 study. The FDR correction with the method by Benjamini-Hochberg was employed to account for multiple comparisons. The multivariable MR analysis was conducted using MendelianRandomization R package in R Software 4.0.2.

### Ethics

This research was conducted using the UK Biobank study under Application Number 66354. The UK Biobank received ethical permits from the Northwest Multi-centre Research Ethics Committee, the National Information Governance Board for Health and Social Care in England and Wales, and the Community Health Index Advisory Group in Scotland. All participants provided written informed consent. The Coordinating Ethics Committee of the Hospital District of Helsinki and Uusimaa (HUS) approved the FinnGen study protocol (number HUS/990/2017). Participants in FinnGen provided informed consent for biobank research on basis of the Finnish Biobank Act. The two-sample MR analysis based on summary-level data from the FinnGen requires no ethical permit.

### Role of funders

None of the funding sources played a role in the study design, data collection, data analyses, interpretation, or writing the manuscript.

## Results

### MR-PheWAS identified 68 clinical outcomes associated with genetic liability to CeD

Table 1 shows the characteristics of 385,917 individuals with a mean age of 56.7 years. A total of 1807 clinical outcomes were defined by the PheCODE schema. After removing the outcomes with the number of cases <120, MR-PheWAS included 1060 distinct phenotypes into 18 disease categories.

**Table 1:** Characteristics of included participants in the UK Biobank.

Characteristic	All participants (n = 385,917)
Age (years), mean (SD)	56.7 (8.0)
Gender, n (%)	
Female	208,227 (54.0)
Male	177,690 (46.0)
BMI (kg/m^2^), n (%)	
Underweight (<18.5)	1995 (0.5)
Normal (18.5–24.9)	126,761 (33.0)
Overweight (25–29.9)	163,400 (42.5)
Obese (≥30)	92,274 (24.0)
Unknown	74 (0.0)
Smoking status, n (%)	
Former or current	176,690 (45.8)
Never	207,296 (53.7)
Unknown	1813 (0.5)
Physical activity^a^, n (%)	
Insufficient	274,605 (71.2)
Sufficient	88,703 (23.0)
Unknown	22,491 (5.8)
Alcohol intake^b^, n (%)	
Excessive intake	104,556 (27.1)
Non/moderate intake	280,506 (72.7)
Unknown	737 (0.2)

BMI, body mass index; SD, standard deviation; TDI, Townsend deprivation index.

a Sufficient physical activity was defined by at least ≥150 min moderate activity per week or ≥75 min vigorous activity per week (or an equivalent combination) according to the 2018 Physical Activity Guidelines for Americans.

b Non/moderate consumption was defined as >0 and ≤ 14 g/day for women; >0 and ≤ 28 g/day for men.

**Fig. 2:**
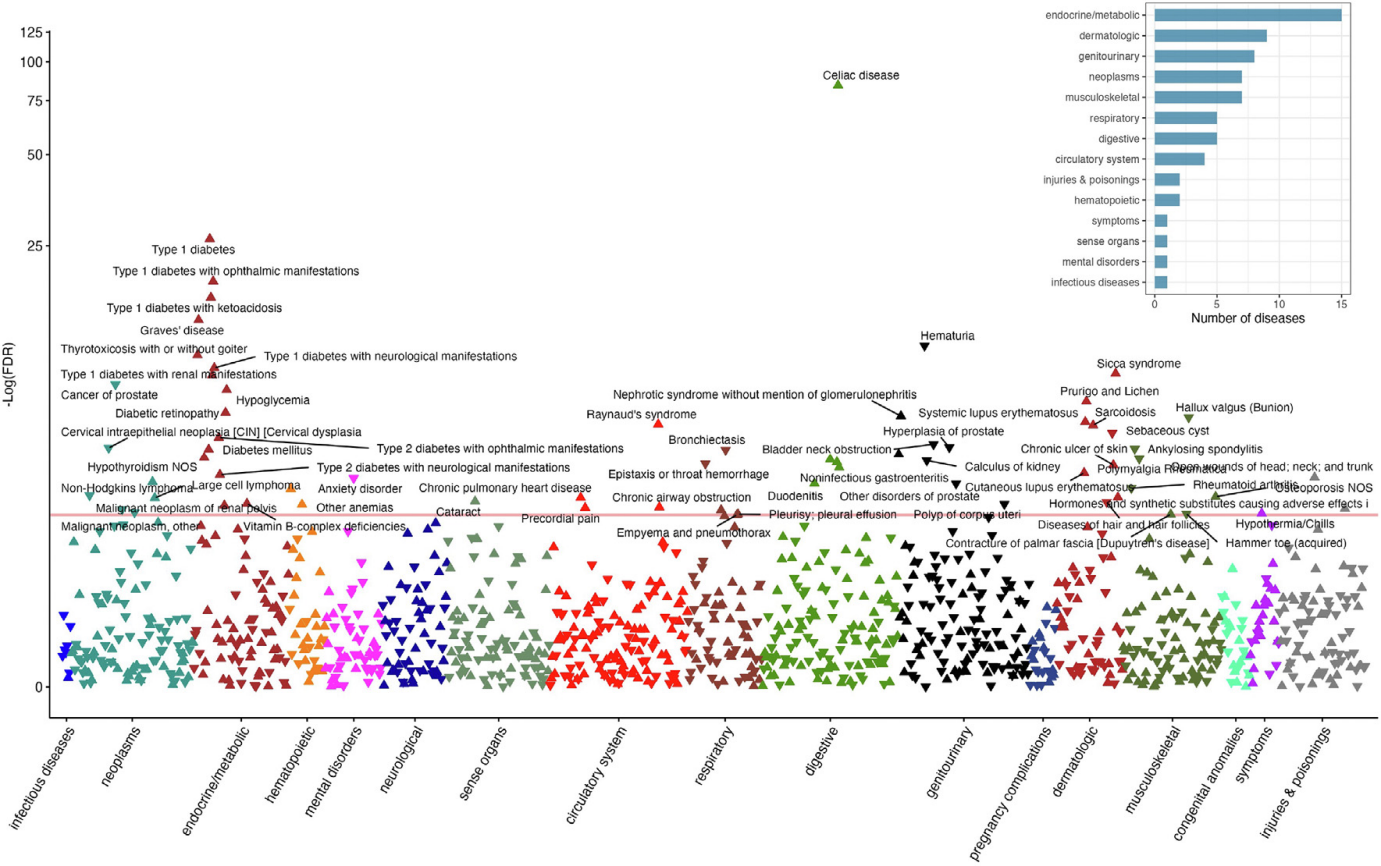
Manhattan plot of the associations between genetic liability to coeliac disease and the risk of 1186 clinical outcomes in the UK Biobank. NOS, Not other specified. The y-axe corresponds to the logarithms of the FDR-adjusted *P* values derived from the phenome-wide Mendelian randomization association analyses. The red line corresponds to the statistical significance level (false discovery rate <0.05). The associations surviving the significance criteria have been annotated. The triangle facing up represents a positive association, otherwise an inverse association. The right-upper corner shows the number of diseases associated with genetic liability to coeliac disease by systems.

Genetically liability to CeD was associated with an increased risk of 47 clinical outcomes and a lower risk of 21 clinical outcomes after multiple testing corrections (Fig. 2 and Supplementary Table S2). Most of the identified outcomes were related to the endocrine/metabolic system (Fig. 2). The strongest association for the CeD polygenic risk score was observed with CeD diagnosis (odds ratio [OR] 1.62, 95% confidence interval [CI] 1.60–1.65; *P* < 9.67 × 10^−85^), which indicates a good validity of used genetic instruments. Several autoimmune diseases were found to be significantly associated with genetic liability to CeD, including type 1 diabetes (T1D; OR 1.09, 95% CI 1.07–1.10; *P* = 5.45 × 10^−27^), Graves' disease (OR 1.06, 95% CI 1.04–1.08; *P* = 2.46 × 10^−11^), Sicca syndrome (known as Sjögren syndrome; OR 1.10, 95% CI 1.07–1.13; *P* = 7.45 × 10^−10^), chronic hepatitis (OR 1.13, 95% CI 1.08–1.18; *P* = 2.40 × 10^−8^), systemic lupus erythematosus (OR 1.10, 95% CI 1.06–1.14; *P* = 8.22 × 10^−7^), sarcoidosis (OR 1.08, 95% CI 1.04–1.11; *P* = 1.22 × 10^−6^), and cutaneous lupus erythematosus (OR 1.12, 95% CI 1.05–1.18; *P* = 1.41 × 10^−4^). In addition, genetic liability to CeD was associated with an elevated risk of non-Hodgkin's lymphoma (OR 1.03, 95% CI 1.01–1.05; *P* = 2.89 × 10^−4^), osteoporosis (OR 1.01, 95% CI 1.00–1.02; *P* = 8.58 × 10^−4^), iron deficiency anaemias (OR 1.01, 95% CI 1.01–1.02; *P* = 4.92 × 10^−4^), and vitamin B-complex deficiencies (OR 1.03, 95% CI 1.01–1.04; *P* = 0.001), and a reduced risk of prostate diseases, including prostatic hyperplasia (OR 0.98, 95% CI 0.97–0.98; *P* = 1.35 × 10^−5^), prostate cancer (OR 0.98, 95% CI 0.98–0.99; *P* = 4.53 × 10^−9^), and other prostate disorders (OR 0.96, 95% CI 0.94–0.98; *P* = 3.31 × 10^−4^).

Most associations did not remain statistically significant in the analysis removing SNPs in the *MHC* gene complex region (Supplementary Table S3); however, the magnitude alternations of the associations differed between outcomes. The associations attenuated for T1D (OR = 1.01; 95% CI 0.80–1.28) and its complications and non-Hodgkin's lymphoma. Nevertheless, the magnitude of the associations for most other identified outcomes increased albeit nonsignificant (e.g., Graves' disease OR = 1.43; 95% CI 0.88–2.31) possibly due to smaller power (a wider CI) caused by reduced variance explained by fewer SNPs. The associations were overall consistent between women and men (Table 2) and age strata (Supplementary Table S4).

**Table 2:** The sex-specific associations between genetic liability to coeliac disease and 68 clinical outcomes in the UK Biobank.

Phecode	Clinical outcome	All participants			Women			Men		
		OR	95% CI	Pval	OR	95% CI	Pval	OR	95% CI	Pval
557.1	Coeliac disease	1.62	1.60–1.65	<5E-324	1.62	1.59–1.66	<5E-324	1.62	1.58–1.67	2.70E-252
250.12	Type 1 diabetes with renal manifestations	1.28	1.18–1.38	1.03E-09	1.18	1.03–1.35	1.92E-02	1.34	1.21–1.48	5.12E-09
250.11	Type 1 diabetes with ketoacidosis	1.21	1.16–1.27	1.81E-17	1.23	1.16–1.31	1.81E-10	1.2	1.12–1.27	1.51E-08
250.14	Type 1 diabetes with neurological manifestations	1.19	1.13–1.26	2.75E-10	1.19	1.09–1.30	8.17E-05	1.19	1.11–1.28	7.94E-07
250.13	Type 1 diabetes with ophthalmic manifestations	1.16	1.13–1.20	1.00E-19	1.19	1.13–1.25	1.54E-11	1.15	1.10–1.20	5.57E-10
242.1	Graves' disease	1.13	1.10–1.17	8.86E-15	1.12	1.08–1.16	6.24E-10	1.19	1.11–1.28	7.58E-07
70.4	Chronic hepatitis	1.13	1.08–1.18	2.40E-08	1.11	1.06–1.17	2.90E-05	1.17	1.08–1.27	1.21E-04
580.12	Non-proliferative glomerulonephritis	1.13	1.07–1.20	2.66E-05	1.08	0.99–1.19	9.34E-02	1.17	1.09–1.27	3.83E-05
695.41	Cutaneous lupus erythematosus	1.12	1.05–1.18	1.41E-04	1.1	1.03–1.17	3.21E-03	1.18	1.04–1.34	8.46E-03
709.2	Sicca syndrome	1.10	1.07–1.13	7.45E-10	1.1	1.06–1.13	9.13E-09	1.12	1.01–1.23	2.49E-02
695.42	Systemic lupus erythematosus	1.10	1.06–1.14	8.22E-07	1.1	1.06–1.15	6.96E-06	1.1	1.00–1.21	4.44E-02
870	Open wounds of head; neck; and trunk	1.10	1.04–1.15	2.04E-04	1.04	0.95–1.13	4.05E-01	1.13	1.06–1.19	8.04E-05
250.1	Type 1 diabetes	1.09	1.07–1.11	4.54E-27	1.12	1.10–1.15	2.09E-21	1.07	1.04–1.09	1.61E-09
580.2	Nephrotic syndrome without mention of glomerulonephritis	1.08	1.05–1.11	4.02E-07	1.12	1.07–1.17	3.33E-06	1.05	1.02–1.09	4.60E-03
697	Sarcoidosis	1.08	1.04–1.11	1.23E-06	1.1	1.05–1.14	1.54E-05	1.06	1.01–1.10	1.23E-02
557	Intestinal malabsorption (non-coeliac)	1.08	1.04–1.12	5.54E-05	1.08	1.04–1.13	5.54E-04	1.07	1.00–1.14	3.57E-02
695.7	Prurigo and Lichen	1.07	1.04–1.10	5.66E-08	1.08	1.04–1.11	1.78E-06	1.06	1.02–1.11	8.08E-03
415.2	Chronic pulmonary heart disease	1.07	1.03–1.12	9.16E-04	1.05	0.99–1.12	9.19E-02	1.09	1.03–1.15	3.17E-03
242	Thyrotoxicosis with or without goitre	1.06	1.04–1.08	2.46E-11	1.06	1.04–1.09	1.11E-09	1.05	1.02–1.09	5.32E-03
962.3	Hormones and synthetic substitutes causing adverse effects in therapeutic use	1.06	1.02–1.09	1.88E-03	1.02	0.98–1.07	2.93E-01	1.09	1.04–1.15	4.64E-04
780	Hypothermia/Chills	1.06	1.02–1.10	2.54E-03	1.04	0.99–1.10	1.31E-01	1.07	1.02–1.12	6.88E-03
251.1	Hypoglycaemia	1.05	1.03–1.07	1.05E-08	1.05	1.02–1.08	3.36E-04	1.05	1.03–1.08	7.94E-06
443.1	Raynaud's syndrome	1.05	1.03–1.06	1.10E-06	1.04	1.02–1.06	1.79E-04	1.05	1.02–1.09	1.57E-03
250.24	Type 2 diabetes with neurological manifestations	1.05	1.02–1.08	1.64E-04	1.1	1.05–1.15	3.30E-05	1.03	1.00–1.06	8.30E-02
250.6	Polyneuropathy in diabetes	1.05	1.02–1.08	1.54E-03	1.09	1.03–1.14	1.60E-03	1.03	1.00–1.07	8.62E-02
443.7	Peripheral angiopathy in diseases classified elsewhere	1.05	1.02–1.08	1.73E-03	1.07	1.01–1.14	3.33E-02	1.04	1.01–1.08	1.62E-02
250.7	Diabetic retinopathy	1.04	1.03–1.06	2.59E-07	1.07	1.04–1.10	1.16E-06	1.03	1.01–1.05	5.63E-03
250.23	Type 2 diabetes with ophthalmic manifestations	1.04	1.02–1.05	4.90E-06	1.05	1.03–1.08	2.71E-05	1.03	1.01–1.05	1.24E-02
202.24	Large cell lymphoma	1.04	1.02–1.07	9.32E-04	1.07	1.03–1.11	1.19E-03	1.03	0.99–1.06	1.15E-01
707	Chronic ulcer of skin	1.03	1.02–1.05	7.21E-05	1.04	1.01–1.06	8.24E-03	1.03	1.01–1.05	3.00E-03
202.2	Non-Hodgkin's lymphoma	1.03	1.01–1.05	2.89E-04	1.04	1.01–1.07	3.12E-03	1.03	1.00–1.05	2.60E-02
709.7	Unspecified diffuse connective tissue disease	1.03	1.01–1.04	8.96E-04	1.03	1.01–1.05	1.22E-02	1.03	1.00–1.05	2.91E-02
261.2	Vitamin B-complex deficiencies	1.03	1.01–1.04	1.37E-03	1.04	1.01–1.06	1.44E-03	1.02	0.99–1.04	1.92E-01
506	Empyema and pneumothorax	1.03	1.01–1.05	2.78E-03	1.01	0.98–1.05	3.87E-01	1.04	1.01–1.06	1.81E-03
535.6	Duodenitis	1.02	1.01–1.03	3.27E-04	1.02	1.01–1.04	1.59E-03	1.01	1.00–1.02	4.21E-02
418.1	Precordial pain	1.02	1.01–1.03	1.81E-03	1.02	1.01–1.04	2.59E-03	1.01	1.00–1.03	1.71E-01
728.71	Contracture of palmar fascia [Dupuytren's disease]	1.02	1.01–1.03	2.69E-03	1.04	1.01–1.06	3.62E-03	1.01	1.00–1.03	7.90E-02
250	Diabetes mellitus	1.01	1.01–1.02	1.66E-05	1.02	1.01–1.03	5.58E-06	1.01	1.00–1.01	7.14E-02
244.4	Hypothyroidism NOS	1.01	1.01–1.02	3.69E-05	1.01	1.00–1.02	1.09E-03	1.02	1.00–1.03	7.91E-03
555	Inflammatory bowel disease and other gastroenteritis and colitis	1.01	1.01–1.02	4.33E-05	1.02	1.01–1.03	1.04E-05	1.01	1.00–1.02	2.51E-01
558	Non-infectious gastroenteritis	1.01	1.01–1.02	8.86E-05	1.02	1.01–1.03	3.51E-05	1.01	1.00–1.02	2.47E-01
280.1	Iron deficiency anaemias, unspecified or not due to blood loss	1.01	1.01–1.02	4.92E-04	1.01	1.00–1.02	4.61E-02	1.02	1.01–1.03	2.62E-03
743.11	Osteoporosis NOS	1.01	1.01–1.02	8.58E-04	1.01	1.00–1.02	2.00E-03	1.01	0.99–1.03	2.10E-01
366	Cataract	1.01	1.00–1.01	1.17E-03	1	1.00–1.01	2.88E-01	1.01	1.01–1.02	2.08E-04
285	Other anaemias	1.01	1.00–1.02	1.45E-03	1.01	1.01–1.02	7.35E-04	1.01	1.00–1.01	2.93E-01
496	Chronic airway obstruction	1.01	1.00–1.02	2.06E-03	1.01	1.00–1.02	1.74E-02	1.01	1.00–1.02	4.47E-02
507	Pleurisy; pleural effusion	1.01	1.00–1.02	2.66E-03	1.01	1.00–1.02	5.49E-02	1.01	1.00–1.02	2.04E-02
300.1	Anxiety disorder	0.99	0.98–0.99	2.16E-04	0.99	0.98–1.00	3.46E-03	0.99	0.97–1.00	2.31E-02
195.1	Malignant neoplasm, other	0.99	0.99–1.00	2.38E-03	0.99	0.98–1.00	5.75E-02	0.99	0.98–1.00	1.76E-02
593	Haematuria	0.98	0.97–0.98	3.61E-12	0.97	0.96–0.98	6.19E-07	0.98	0.97–0.99	1.03E-06
185	Cancer of prostate	0.98	0.97–0.98	4.53E-09	–	–	–	0.98	0.97–0.98	4.53E-09
706.2	Sebaceous cyst	0.98	0.97–0.99	2.97E-06	0.98	0.97–1.00	1.28E-02	0.97	0.96–0.99	3.22E-05
600	Hyperplasia of prostate	0.98	0.98–0.99	1.35E-05	–	–	–	0.98	0.98–0.99	1.35E-05
714.1	Rheumatoid arthritis	0.98	0.97–0.99	4.65E-04	0.99	0.97–1.00	7.19E-02	0.97	0.95–0.99	4.36E-04
704	Diseases of hair and hair follicles	0.98	0.97–0.99	1.24E-03	0.99	0.97–1.00	1.23E-01	0.97	0.96–0.99	2.47E-03
622.1	Polyp of corpus uteri	0.98	0.97–0.99	1.42E-03	0.98	0.97–0.99	1.42E-03	–	–	–
735.3	Hallux valgus (Bunion)	0.97	0.96–0.98	4.72E-07	0.98	0.97–0.99	4.03E-05	0.94	0.91–0.98	4.89E-04
180.3	Cervical intraepithelial neoplasia [CIN] [Cervical dysplasia]	0.97	0.96–0.99	1.43E-05	0.97	0.96–0.99	1.43E-05	–	–	–
496.3	Bronchiectasis	0.97	0.96–0.98	1.74E-05	0.97	0.95–0.98	1.35E-04	0.98	0.96–1.00	3.02E-02
717	Polymyalgia Rheumatica	0.97	0.95–0.98	4.06E-05	0.96	0.94–0.98	1.43E-04	0.98	0.95–1.00	7.11E-02
594.1	Calculus of kidney	0.97	0.96–0.99	4.82E-05	0.97	0.94–0.99	2.63E-03	0.98	0.96–0.99	4.54E-03
477	Epistaxis or throat haemorrhage	0.97	0.96–0.98	6.42E-05	0.98	0.96–1.00	5.56E-02	0.96	0.95–0.98	2.92E-04
735.21	Hammer toe (acquired)	0.97	0.96–0.99	2.61E-03	0.98	0.96–1.00	1.65E-02	0.95	0.91–1.00	4.24E-02
596.1	Bladder neck obstruction	0.96	0.94–0.98	9.87E-06	0.97	0.88–1.07	5.51E-01	0.96	0.94–0.98	1.15E-05
602	Other disorders of prostate	0.96	0.94–0.98	3.31E-04	–	–	–	0.96	0.94–0.98	3.31E-04
715.2	Ankylosing spondylitis	0.93	0.89–0.96	1.55E-05	0.96	0.91–1.02	1.85E-01	0.91	0.87–0.95	1.24E-05
159.2	Malignant neoplasm of small intestine, including duodenum	0.92	0.88–0.97	7.76E-04	0.93	0.87–1.00	4.33E-02	0.92	0.86–0.98	6.96E-03
189.12	Malignant neoplasm of renal pelvis	0.90	0.84–0.96	1.96E-03	0.88	0.79–0.99	3.02E-02	0.9	0.83–0.99	2.54E-02

CI, confidence interval; OR, odds ratio.

### Two-sample MR replicated 38 associations

**Fig. 3:**
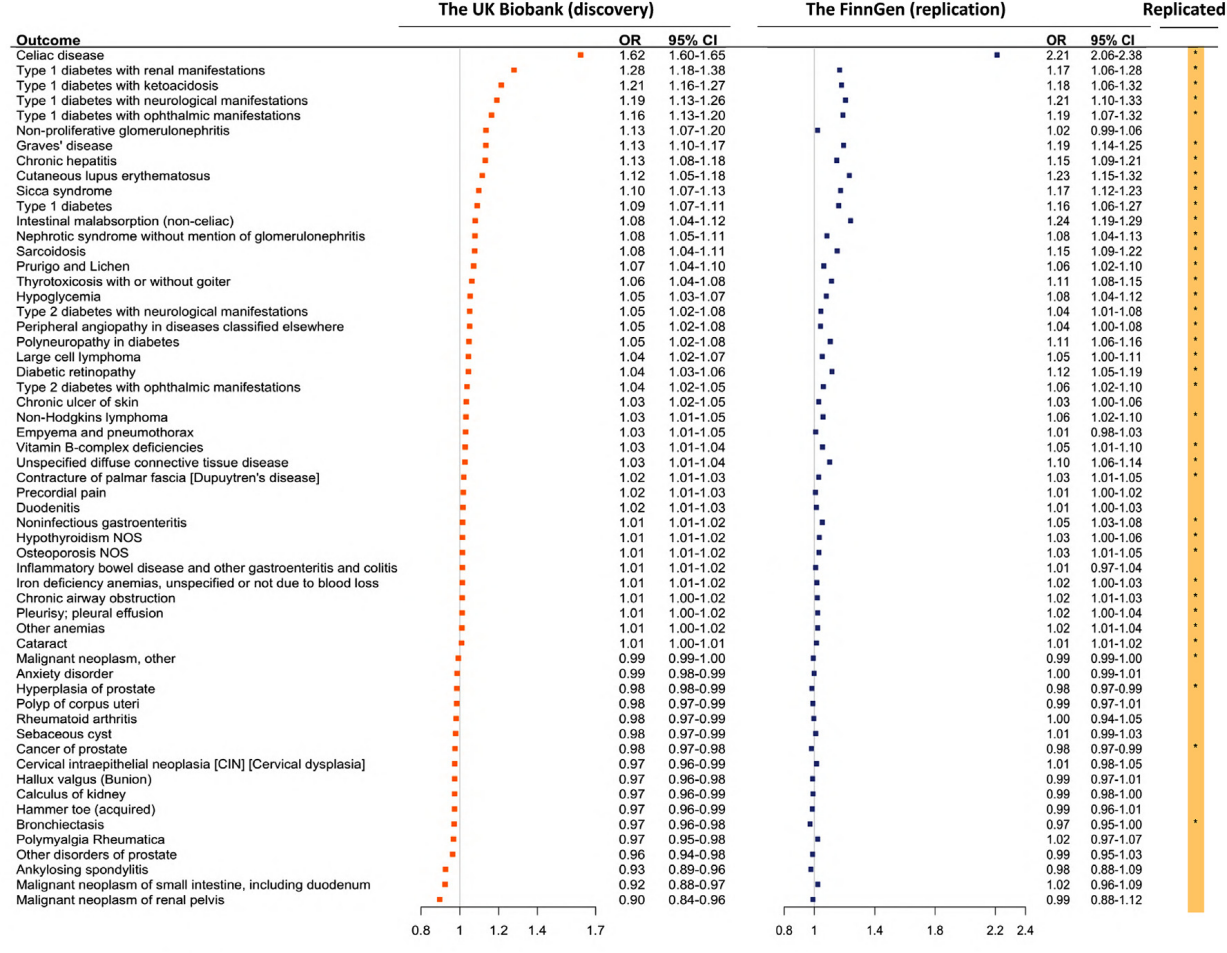
The identified associations for genetic liability to coeliac disease in the UK Biobank and replication in the FinnGen study. CI, confidence interval; OR, odds ratio. The associations in the FinnGen study were estimated using the inverse variance weighted method. The associations replicated at the *P* value < 0.05 were marked by a start (∗) sign in the right column.

Of the 68 outcomes associated with CeD in the UK Biobank, 57 were available in the FinnGen study for the replication analysis (Supplementary Table S5). Among 57, genetic liability to CeD was associated with an increased risk of 34 clinical outcomes and a reduced risk of 4 outcomes at the nominal significance level (*P* < 0.05; Fig. 3). Likewise, the strongest association was observed for the CeD diagnosis (OR 2.21, 95% CI 2.06–2.38; *P* = 2.02 × 10^−103^). The associations for identified autoimmune diseases, non-Hodgkin's lymphoma, osteoporosis, cataract, malnutrition (iron deficiency anaemias and vitamin B-complex deficiencies), and prostate diseases were replicated. Moderate-to-high heterogeneity was observed between SNP estimates for many outcomes (Supplementary Table S6). However, we did not observe a significant indication of directional pleiotropy detected by the MR-Egger intercept test (Supplementary Table S6), except for 6 outcomes (*P* for MR-Egger intercept <0.05). The MR-PRESSO analysis detected SNP outliers for many associations; however, these associations including the associations for 6 outcomes detected by MR-Egger intercept test remained after the removal of identified outliers (Supplementary Table S6).

### Multivariable MR

Using the inverse variance weighted method with fixed effects, genetically predicted abundance of *genus Bifidobacterium* was associated with osteoporosis (*P* = 0.019) and possibly with lupus erythematosus (*P* = 0.061) after FDR correction (Supplementary Table S7). The associations between genetic liability to CeD and the risk of osteoporosis and cutaneous lupus erythematosus did not change after adjustment for predicted abundance of *genus Bifidobacterium* (Supplementary Table S8), indicating limited chance of the second assumption of MR analysis being violated in our study.

## Discussion

### Main findings

This MR-PheWAS explored the clinical diseases associated with genetic liability to CeD in a comprehensive way in two large-scale population-based cohorts. A strong association between genetic liability to CeD and actual CeD diagnosis was detected, which indicates a good validity of the used genetic instruments. In addition, genetic liability to CeD was associated with 68 clinical outcomes in the UK Biobank study, and 38 associations were replicated in the FinnGen, including a higher risk of several autoimmune diseases (type 1 diabetes and its complications, Graves' disease, Sjögren syndrome, chronic hepatitis, systemic and cutaneous lupus erythematosus, and sarcoidosis), non-Hodgkin's lymphoma, osteoporosis, and malnutrition, and a lower risk of prostate diseases. The associations between genetic liability to CeD and autoimmune diseases are in line with a recent two-sample MR study.^35^ Furthermore, we found that these associations were consistent between women and men and different age strata if available. In the secondary analysis, the associations for T1D and its complications and non-Hodgkin's lymphoma largely attenuated when excluding SNPs in the *MHC* region; however, the associations for other identified outcomes appeared to be increased albeit nonsignificant, possibly due to inadequate power.

### CeD and T1D

We found a strong association between genetic liability to CeD and T1D. This association confirms earlier data of an increased risk of T1D both before and after CeD.^36^^,^^37^ In 2014, our meta-analysis based on pooled data from 26,000 individuals with T1D found a prevalence of biopsy-verified CeD of 6.0%,^36^ and most T1D expert committees recommend screening for CeD.^38^^,^^39^ Both CeD and T1D are characterized by the early appearance of disease-specific antibodies and share a strong link to *HLA* class II. In fact, one study suggests that the mere presence of *HLA-DQ2* increases the risk of T1D 3.5-fold^40^ which is more than the future risk of T1D in a Swedish CeD cohort (hazard ratio = 2.4).^37^ Contrasting features of the two diseases include the presence of a known necessary trigger, a female predominance, and a high prevalence of homozygosity for *HLA DR3-DQ2* in CeD.^41^ While these two diseases clearly share a common genetic liability, environmental risk factors are likely to differ, and potentially not only gluten but also other factors as revealed by the so/called “coeliac epidemic” in Sweden,^42^^,^^43^ where nothing similar has been seen in type 1 diabetes. Of note, this association did not persist in the analysis removing genetic instruments in the *MHC* region, which indicates that this association may be heavily driven by this shared pleiotropic gene on overall autoimmunity. Even though this finding may undermine the causality of this MR association by showing that this analysis may be possibly violating the third assumption, it suggests clinical complications as discussed above. Additionally, in our study, CeD was particularly associated with T1D complications, and some studies have indeed suggested that CeD may complicate T1D.^44^^,^^45^ These data may suggest that patients with CeD and T1D should undergo closer monitoring for T1D complications. However, it is possible that patients with T1D with complications are more likely to be screened for additional disorders such as CeD resulting in ascertainment bias.

### CeD and thyroid disease

Grave's disease, and to a lesser extent thyrotoxicosis and hypothyroidism (often autoimmune) were associated with CeD. The European Society for the Study of Coeliac Disease (ESsCD) stipulates that patients with Grave's disease and Hashimoto's disease should be screened for CeD.^46^ We have previously shown that individuals with an inpatient diagnosis of CeD were at a 2.9-fold increased risk of future hyperthyroidism, and at a 4.4-fold increased risk of hypothyroidism in a nationwide Swedish population.^47^ Meta-analyses reviewing CeD among patients with autoimmune thyroid disease,^48^ and vice versa^49^ have shown increased prevalence of both diseases.

### CeD and other autoimmune diseases

Strong associations with a genetic liability of CD were also seen for chronic hepatitis, lupus erythematosus, and sarcoidosis; findings which are in line with earlier research (liver,^50^^,^^51^ lupus erythematous,^52^ and sarcoidosis^53^). Of note, an earlier MR study by Inamo et al. have already reported an association between CeD and systemic lupus erythematosus.^54^ While we found a positive association with the Sicca syndrome (Sjogren's syndrome), earlier reports in this field have been contradictory. One review suggests that patients with Sicca syndrome be screened for CeD but not the other way around.^55^

### CeD and other disorders

Our investigation revealed associations with several well-known complications of genetic liability to CeD that are non-Hodgkin's lymphoma, osteoporosis, and iron deficiency anaemia. While absolute risks of non-Hodgkin's lymphoma are low (one earlier study reported 70.3 vs 26.2 per 100,000 person-years in the general population, equivalent to one extra case per 227 coeliac patients followed-up for ten years), it has nevertheless been strongly linked to CeD,^56^ especially among patients with persistent villus atrophy at control biopsy.^57^ The attenuated association in the secondary analysis removing SNPs in *MHC* region may indicate a shared genetic aetiology behind this link. Additionally, refractory CeD, defined as persistent villus atrophy and malabsorption despite adherence to a gluten-free diet for more than one year,^58^ can be a precursor to enteropathy-associated T cell lymphoma, a subtype of non-Hodgkin's lymphoma that carries a poor prognosis.^59^ The ESsCD recommends screening for CeD in both iron deficiency anaemia and unexplained osteoporosis.^46^ These recommendations are supported by two earlier meta-analyses.^60^^,^^61^ Up to 1 in 30 patients with iron-deficiency anaemia may suffer from CeD, often undiagnosed.^61^ Malabsorption, a feature of classical CeD, could probably also explain the association with vitamin B deficiencies that we detected.

### Novel and contradictory findings

We noted an association with Dupuytren's disease. To our knowledge, this has not been reported before. In addition, genetic liability to CeD seemed to be inversely related to one particular group of diseases, those affecting the prostate. Research in this field has been scarce, but in 2012, we noted a (non-significant) decrease in the risk of prostate cancer among 11,000 men with biopsy-verified CeD (hazard ratio = 0.92; 95% CI 0.79–1.08).^62^ The current study found no associations with Addison's diseases, unexplained ataxia, psoriasis, IgA nephropathy, infertility, and various syndromes such as Down's and Turner's syndrome. These have all been linked to CeD in earlier observational studies. Some of these conditions are rare and thus were removed in the UK Biobank analysis due to few cases, while others such as infertility may not be truly associated with CeD.^63^^,^^64^

### Mechanisms

Regarding CeD and T1D and its complications, the shared *HLA* genotypes may be a dominant underlying mechanism,^41^ which is supported by our secondary analysis where the associations largely attenuated after the removal of genetic instruments in the *MHC* region. Of note, this analysis removed all SNPs in *MHC*, a much wider region than *HLA-DQ2* and *HLA-DQ8*, in a conservative way to reduce the influence of high linkage disequilibrium in this gene region. Thus, this analysis may reflect the effects of not only CeD-related *HLA* genes but the overall role of the whole *HLA* complex. In addition, non-*HLA*-related pathways are also important.^65^ For example, non-*HLA* CeD-related loci have been found to be enriched in genes predicted to control T cell activation and B cell help.^65^ These pathways play vital roles in interleukin regulation and thus may also explain the associations of CeD with its causal comorbidities. According to an increased magnitude of some associations in the analysis after excluding *MHC* SNPs, the non-*HLA* pathways seem to be more important for these outcomes, which warrants further verification.

### Clinical implications

Our study supports the current recommendations to reinforce comorbidity management and coeliac awareness among patients with CeD, in particular for T1D and autoimmune thyroid disease.^66^, ^67^, ^68^ Diagnosing CeD in e.g., T1D may potentially influence the prognosis of the latter. Our study also confirms the associations with several additional autoimmune conditions sometimes screened for CeD, sometimes not. While evidence that early detection of CeD may impact the long-term risk of additional autoimmunity is scarce,^69^ this cannot be ruled out, and it seems that early treatment of CeD (conditional on early diagnosis) and mucosal healing are inversely related to certain fractures^57^ and the development of non-Hodgkin's lymphoma.^57^ Early coeliac diagnosis is likely to attenuate any malabsorption, with positive effects on e.g., iron deficiency anaemia upon mucosal healing of a previously undiagnosed CeD.

### Limitations

The study has several advantages, including the MR design that strengthens causal inference, an exploration of the associations of CeD with a wide range of diseases, a replication in an independent study, and consistent results from sensitivity analyses. Limitations need to be discussed when interpreting our findings. First, the magnitude of the associations in this study appeared smaller than that in observational studies even though this should not bias causal inference. This discrepancy may be attributed to two major reasons: 1) the exposure in this study is the genetic liability to CeD instead of the actual diagnosis that is usually used in observational studies; and 2) we mimicked the lifetime exposure to CeD since birth and thus estimated the lifetime risk of outcomes caused by CeD in this MR study; however, a large part of patients with CeD often get a delayed diagnosis, which may substantially increase the risk of other disorders in observational studies. Second, rare outcomes associated with CeD might be removed or overlooked in the analysis due to few cases. Third, the associations of genetic liability to CeD with type 1 diabetes and non-Hodgkin's lymphoma attenuated when excluding SNPs in the extended *MHC* region, indicating shared genetic aetiology and potential violation of the third assumption of MR. However, this finding should not compromise the clinical complications on comorbidities. Fourth, we might have inadequate power due to a much smaller variance explained by the used instrumental variable in the secondary analysis where *MHC* SNPs were removed. Fifth, we could not completely rule out horizontal pleiotropic effects even though limited indication of this bias was from MR-Egger or MR-PRESSO analyses. Finally, clinical outcomes were majorly defined by inpatient hospital records, which may overlook mild diseases that do not need hospitalization.

### Conclusions

This MR-PheWAS study found a wide range of clinical outcomes, in particular autoimmune diseases, non-Hodgkin's lymphoma, osteoporosis, and malnutrition, associated with CeD. *HLA* genotypes appeared to be dominantly important for the associations of CeD with T1D and non-Hodgkin's lymphoma. These findings reveal comorbidities of CeD and suggest the necessity of comorbidity monitoring among this population. Further studies illuminating the mechanisms behind coeliac comorbidity are needed.

## Contributors

S.Y., F.J., and X.L had full access to all the data in the study and take responsibility for the integrity of the data and the accuracy of the data analysis. S.Y. and J.F.L. conceived and designed the study. S.Y. and F.J. undertook the statistical analyses. S.Y. made figures. S.Y. and J.F.L. wrote the first draft of the manuscript. S.Y., F.J., J.C., B.L., P.H.G., D.L., S.C.L., X.L., and J.F.L. made critical revision of the manuscript for important intellectual contents. All authors read and approved the final version of the manuscript.

## Data sharing statement

All data will be available to approved users of the UK Biobank upon application. The summary-level data from the FinnGen are publicly available (https://finngen.gitbook.io/documentation/).

## Declaration of interests

Dr. Leffler is an employee of Takeda. Dr. Ludvigsson has coordinated an unrelated study for the Swedish IBD quality register (SWIBREG). That study received funding from Janssen Corporation. Dr. Ludvigsson has also received financial support from MSD, developing a paper reviewing national healthcare registers in China. Dr. Ludvigsson has an ongoing research collaboration with Takeda. The other authors report no conflicts of interest.
