# Supplementary material for: Phenome-wide Mendelian randomization analysis reveals multiple health comorbidities of coeliac disease
Source: eBioMedicine. 2024 Feb 21;101:105033. doi: 10.1016/j.ebiom.2024.105033 (PMC10900254; doi:10.1016/j.ebiom.2024.105033)
Supplement: Supplementary Figure and Tables S1–S8 [file mmc1.docx]

Supporting information for

**Phenome-wide Mendelian randomization analysis reveals multiple health consequences of coeliac disease**

Shuai Yuan, Fangyuan Jiang, Jie Chen, Benjamin Lebwohl, Peter HR Green, Daniel Leffler, Susanna C. Larsson, Xue Li, Jonas F. Ludvigsson

Table of Contents

[Supplementary Table 1. Genetic instruments for coeliac disease 2](#_Toc157351641)

[Supplementary Table 2. Top 200 associations between genetic liability to coeliac disease and the risk of clinical outcomes in the UK Biobank 3](#_Toc157351642)

[Supplementary Table 3. The associations between genetic liability to coeliac disease and 68 clinical outcomes using genetic instruments including and excluding MHC SNPs. 7](#_Toc157351643)

[Supplementary Table 4. The associations between genetic liability to coeliac disease and 68 clinical outcomes by age stratification 9](#_Toc157351644)

[Supplementary Table 5. Data sources for 57 outcomes in the FinnGen study R9 11](#_Toc157351645)

[Supplementary Table 6. The associations between genetic liability to coeliac disease and 57 outcomes in the primary and sensitivity Mendelian randomization analyses in FinnGen 13](#_Toc157351646)

[Supplementary Table 7. Genetically predicted abundance of genus Bifidobacterium in relation to CeD-associated outcomes 15](#_Toc157351647)

[Supplementary Table 8. Univariable (UVMR) and multivariable MR (MVMR) analysis on the associations of genetic liability to CeD with osteoporosis and cutaneous lupus erythematosus 17](#_Toc157351648)

[Supplementary Figure 1. Flow chart of study population selection and sample quality control in the UK Biobank. 18](#_Toc157351649)

# **Supplementary Table 1.** Genetic instruments for coeliac disease

| **SNP** | **EA** | **EAF** | **Beta** | **SE** | ***Pval*** | **Nearby gene** | **xMHC** | ***F* statistic** |
| --- | --- | --- | --- | --- | --- | --- | --- | --- |
| rs4445406 | C | 0.33 | -0.14 | 0.02 | 5.42E-12 | MMEL1 | FALSE | 47.5 |
| rs12068671 | C | 0.20 | -0.16 | 0.02 | 1.40E-10 | SLC25A38P1 | FALSE | 41.2 |
| rs11801183 | T | 0.82 | -0.14 | 0.02 | 1.69E-08 | RP1-15D23.2 | FALSE | 31.8 |
| rs1323292 | A | 0.18 | 0.26 | 0.03 | 4.23E-25 | RGS1 | FALSE | 107.1 |
| rs10800746 | T | 0.71 | -0.11 | 0.02 | 2.57E-08 | C1orf106 | FALSE | 31.0 |
| rs13003464 | G | 0.64 | 0.15 | 0.02 | 4.34E-16 | PUS10 | FALSE | 66.1 |
| rs990171 | C | 0.78 | -0.18 | 0.02 | 1.22E-16 | SLC9A4 | FALSE | 68.6 |
| rs1018326 | C | 0.56 | 0.15 | 0.02 | 3.06E-16 | LINC01934 | FALSE | 66.8 |
| rs6715106 | G | 0.95 | -0.24 | 0.04 | 8.38E-09 | STAT4 | FALSE | 33.2 |
| rs1980422 | T | 0.26 | -0.17 | 0.02 | 1.43E-15 | CD28 | FALSE | 63.7 |
| rs7616215 | T | 0.36 | -0.11 | 0.02 | 7.27E-09 | CCR3 | FALSE | 33.5 |
| rs2097282 | T | 0.31 | -0.18 | 0.02 | 1.13E-20 | TKT | FALSE | 86.9 |
| rs61579022 | A | 0.39 | 0.11 | 0.02 | 9.92E-09 | ARHGAP31 | FALSE | 32.9 |
| rs76830965 | A | 0.08 | 0.31 | 0.03 | 2.57E-27 | IL12A-AS1 | FALSE | 117.2 |
| rs10513548 | C | 0.90 | -0.19 | 0.03 | 4.23E-08 | IL12A-AS1 | FALSE | 30.0 |
| rs2030519 | A | 0.47 | 0.28 | 0.02 | 3.00E-49 | LPP | FALSE | 217.6 |
| rs13132308 | G | 0.84 | -0.35 | 0.03 | 1.87E-38 | IL21-AS1 | FALSE | 168.2 |
| rs1050976 | T | 0.52 | -0.11 | 0.02 | 1.84E-09 | IRF4 | FALSE | 36.1 |
| rs13195040 | G | 0.93 | 1.12 | 0.03 | 1.00E-200 | ZNF184 | TRUE | 1479.7 |
| rs16894681 | G | 0.02 | -0.36 | 0.06 | 3.24E-08 | XXbac-BPG308J9.3 | TRUE | 30.6 |
| rs2523776 | G | 0.89 | -0.39 | 0.03 | 2.20E-31 | TBC1D22B | TRUE | 135.8 |
| rs57035982 | A | 0.04 | -0.23 | 0.04 | 2.26E-08 | TBC1D22B | TRUE | 31.3 |
| rs9378221 | C | 0.18 | -0.50 | 0.03 | 3.07E-71 | TRIM26 | TRUE | 318.5 |
| rs11753326 | C | 0.09 | -0.52 | 0.04 | 1.47E-47 | SFTA2 | TRUE | 209.9 |
| rs3871466 | C | 0.85 | -0.76 | 0.03 | 1.72E-114 | MUC22 | TRUE | 517.2 |
| rs9380236 | G | 0.91 | -0.51 | 0.04 | 2.95E-40 | HLA-B | TRUE | 176.4 |
| rs2229092 | C | 0.94 | -0.70 | 0.05 | 5.41E-52 | LTA | TRUE | 230.2 |
| rs2509217 | T | 0.96 | -0.74 | 0.07 | 5.75E-28 | TBC1D22B | TRUE | 120.2 |
| rs17421624 | C | 0.33 | -0.93 | 0.02 | 1.00E-200 | TNXB | TRUE | 1479.7 |
| rs9267873 | T | 0.40 | 1.08 | 0.03 | 1.00E-200 | TBC1D22B | TRUE | 1479.7 |
| rs3129882 | A | 0.55 | 0.82 | 0.02 | 1.00E-200 | HLA-DRA | TRUE | 1479.7 |
| rs2071473 | T | 0.39 | -0.79 | 0.02 | 1.00E-200 | XXbac-BPG246D15.9 | TRUE | 1257.9 |
| rs991760 | A | 0.94 | -0.48 | 0.04 | 1.23E-29 | PSMB9 | TRUE | 127.8 |
| rs6934214 | A | 0.04 | -0.58 | 0.06 | 1.22E-21 | HLA-DMB | TRUE | 91.3 |
| rs9276969 | A | 0.03 | 0.38 | 0.06 | 6.47E-11 | HLA-DOA | TRUE | 42.7 |
| rs13203715 | G | 0.89 | -0.36 | 0.03 | 1.21E-31 | TBC1D22B | TRUE | 137.0 |
| rs6913838 | C | 0.42 | -0.38 | 0.02 | 1.10E-83 | TBC1D22B | TRUE | 375.7 |
| rs2179920 | T | 0.23 | 0.67 | 0.02 | 1.00E-200 | HLA-DPB1 | TRUE | 1001.0 |
| rs3130014 | G | 0.34 | 0.31 | 0.02 | 1.20E-59 | TBC1D22B | TRUE | 265.3 |
| rs9469591 | C | 0.12 | 0.46 | 0.03 | 1.10E-72 | TBC1D22B | FALSE | 325.1 |
| rs205281 | C | 0.98 | 0.42 | 0.06 | 9.84E-13 | TBC1D22B | FALSE | 50.9 |
| rs55743914 | T | 0.77 | 0.19 | 0.02 | 1.14E-18 | PTPRK | FALSE | 77.8 |
| rs17264332 | G | 0.83 | 0.25 | 0.02 | 4.98E-30 | RP11-95M15.1 | FALSE | 129.6 |
| rs182429 | G | 0.56 | -0.15 | 0.02 | 8.49E-16 | TAGAP | FALSE | 64.8 |
| rs1107943 | C | 0.94 | 0.20 | 0.03 | 7.95E-09 | RP1-155D22.1 | FALSE | 33.3 |
| rs79758729 | G | 0.89 | 0.16 | 0.03 | 2.12E-08 | ELMO1 | FALSE | 31.4 |
| rs744254 | A | 0.73 | 0.12 | 0.02 | 3.04E-08 | RP11-563J2.3 | FALSE | 30.7 |
| rs1250552 | G | 0.44 | -0.15 | 0.02 | 7.97E-17 | ZMIZ1 | FALSE | 69.4 |
| rs7104791 | C | 0.79 | -0.15 | 0.02 | 1.89E-11 | COLCA2 | FALSE | 45.1 |
| rs10892258 | A | 0.23 | -0.15 | 0.02 | 1.73E-11 | AP002954.4 | FALSE | 45.3 |
| rs61907765 | T | 0.24 | 0.16 | 0.02 | 3.43E-13 | ETS1 | FALSE | 52.9 |
| rs3184504 | C | 0.54 | -0.18 | 0.02 | 5.42E-21 | SH2B3 | FALSE | 88.4 |
| rs11851414 | C | 0.21 | 0.12 | 0.02 | 4.71E-08 | ZFP36L1 | FALSE | 29.8 |
| rs1378938 | C | 0.32 | -0.12 | 0.02 | 7.79E-09 | CSK | FALSE | 33.3 |
| rs6498114 | T | 0.24 | -0.13 | 0.02 | 5.83E-10 | CIITA | FALSE | 38.4 |
| rs11875687 | C | 0.14 | 0.16 | 0.03 | 1.92E-10 | PTPN2 | FALSE | 40.5 |
| rs1893592 | C | 0.72 | -0.12 | 0.02 | 2.96E-09 | UBASH3A | FALSE | 35.2 |
| rs4821124 | C | 0.18 | 0.15 | 0.02 | 5.72E-11 | YDJC | FALSE | 42.9 |

EA, effect allele; EAF, effect allele frequency; SNP, single nucleotide polymorphism.

# **Supplementary Table 2.** Top 200 associations between genetic liability to coeliac disease and the risk of clinical outcomes in the UK Biobank

| **Phecode** | **Clinical outcome** | **System** | **Cases** | **Controls** | **OR** | **95% CI** | ***Pval*** |
| --- | --- | --- | --- | --- | --- | --- | --- |
| 557.1 | Coeliac disease | digestive | 2715 | 276715 | 1.62 | 1.6-1.65 | 0.00E+00 |
| 250.1 | Type 1 diabetes | endocrine/metabolic | 3441 | 350991 | 1.09 | 1.07-1.11 | 4.54E-27 |
| 250.13 | Type 1 diabetes with ophthalmic manifestations | endocrine/metabolic | 749 | 350991 | 1.16 | 1.13-1.2 | 1.00E-19 |
| 250.11 | Type 1 diabetes with ketoacidosis | endocrine/metabolic | 390 | 350991 | 1.21 | 1.16-1.27 | 1.81E-17 |
| 242.1 | Graves' disease | endocrine/metabolic | 800 | 357107 | 1.13 | 1.1-1.17 | 8.86E-15 |
| 695.21 | Dermatitis herpetiformis | dermatologic | 85 | 373643 | 1.42 | 1.29-1.56 | 1.33E-13 |
| 593 | Haematuria | genitourinary | 20352 | 342565 | 0.98 | 0.97-0.98 | 3.61E-12 |
| 242 | Thyrotoxicosis with or without goitre | endocrine/metabolic | 2726 | 357107 | 1.06 | 1.04-1.08 | 2.46E-11 |
| 250.14 | Type 1 diabetes with neurological manifestations | endocrine/metabolic | 266 | 350991 | 1.19 | 1.13-1.26 | 2.75E-10 |
| 709.2 | Sicca syndrome | dermatologic | 935 | 369073 | 1.10 | 1.07-1.13 | 7.45E-10 |
| 250.12 | Type 1 diabetes with renal manifestations | endocrine/metabolic | 122 | 350991 | 1.28 | 1.18-1.38 | 1.03E-09 |
| 185 | Cancer of prostate | neoplasms | 14185 | 349822 | 0.98 | 0.97-0.98 | 4.53E-09 |
| 251.1 | Hypoglycaemia | endocrine/metabolic | 2735 | 346140 | 1.05 | 1.03-1.07 | 1.05E-08 |
| 70.4 | Chronic hepatitis | infectious diseases | 429 | 376556 | 1.13 | 1.08-1.18 | 2.40E-08 |
| 695.7 | Prurigo and Lichen | dermatologic | 1364 | 373643 | 1.07 | 1.04-1.1 | 5.66E-08 |
| 250.7 | Diabetic retinopathy | endocrine/metabolic | 3176 | 356134 | 1.04 | 1.03-1.06 | 2.59E-07 |
| 580.2 | Nephrotic syndrome without mention of glomerulonephritis | genitourinary | 987 | 353615 | 1.08 | 1.05-1.11 | 4.02E-07 |
| 735.3 | Hallux valgus (Bunion) | musculoskeletal | 8083 | 364490 | 0.97 | 0.96-0.98 | 4.72E-07 |
| 695.42 | Systemic lupus erythematosus | dermatologic | 555 | 372042 | 1.10 | 1.06-1.14 | 8.22E-07 |
| 443.1 | Raynaud's syndrome | circulatory system | 2680 | 367618 | 1.05 | 1.03-1.06 | 1.10E-06 |
| 697 | Sarcoidosis | dermatologic | 937 | 373643 | 1.08 | 1.04-1.11 | 1.23E-06 |
| 706.2 | Sebaceous cyst | dermatologic | 10435 | 373939 | 0.98 | 0.97-0.99 | 2.97E-06 |
| 250.23 | Type 2 diabetes with ophthalmic manifestations | endocrine/metabolic | 3539 | 350991 | 1.04 | 1.02-1.05 | 4.90E-06 |
| 596.1 | Bladder neck obstruction | genitourinary | 2636 | 363549 | 0.96 | 0.94-0.98 | 9.87E-06 |
| 600 | Hyperplasia of prostate | genitourinary | 22272 | 349822 | 0.98 | 0.98-0.99 | 1.35E-05 |
| 180.3 | Cervical intraepithelial neoplasia [CIN] [Cervical dysplasia] | neoplasms | 6180 | 347009 | 0.97 | 0.96-0.99 | 1.43E-05 |
| 715.2 | Ankylosing spondylitis | musculoskeletal | 759 | 369947 | 0.93 | 0.89-0.96 | 1.55E-05 |
| 250 | Diabetes mellitus | endocrine/metabolic | 32534 | 350991 | 1.01 | 1.01-1.02 | 1.66E-05 |
| 496.3 | Bronchiectasis | respiratory | 4962 | 333410 | 0.97 | 0.96-0.98 | 1.74E-05 |
| 580.12 | Non-proliferative glomerulonephritis | genitourinary | 232 | 353615 | 1.13 | 1.07-1.2 | 2.66E-05 |
| 244.4 | Hypothyroidism NOS | endocrine/metabolic | 23427 | 357107 | 1.01 | 1.01-1.02 | 3.69E-05 |
| 717 | Polymyalgia Rheumatica | musculoskeletal | 3558 | 382241 | 0.97 | 0.95-0.98 | 4.06E-05 |
| 555 | Inflammatory bowel disease and other gastroenteritis and colitis | digestive | 24223 | 276715 | 1.01 | 1.01-1.02 | 4.33E-05 |
| 594.1 | Calculus of kidney | genitourinary | 5104 | 372991 | 0.97 | 0.96-0.99 | 4.82E-05 |
| 557 | Intestinal malabsorption (non-coeliac) | digestive | 606 | 276715 | 1.08 | 1.04-1.12 | 5.54E-05 |
| 477 | Epistaxis or throat haemorrhage | respiratory | 4161 | 358682 | 0.97 | 0.96-0.98 | 6.42E-05 |
| 707 | Chronic ulcer of skin | dermatologic | 3250 | 382298 | 1.03 | 1.02-1.05 | 7.21E-05 |
| 558 | Non-infectious gastroenteritis | digestive | 18609 | 276715 | 1.01 | 1.01-1.02 | 8.86E-05 |
| 695.41 | Cutaneous lupus erythematosus | dermatologic | 255 | 372042 | 1.12 | 1.05-1.18 | 1.41E-04 |
| 250.24 | Type 2 diabetes with neurological manifestations | endocrine/metabolic | 1295 | 350991 | 1.05 | 1.02-1.08 | 1.64E-04 |
| 870 | Open wounds of head; neck; and trunk | injuries & poisonings | 349 | 371072 | 1.10 | 1.04-1.15 | 2.04E-04 |
| 300.1 | Anxiety disorder | mental disorders | 17971 | 305591 | 0.99 | 0.98-0.99 | 2.16E-04 |
| 202.2 | Non-Hodgkin’s lymphoma | neoplasms | 2982 | 376925 | 1.03 | 1.01-1.05 | 2.89E-04 |
| 535.6 | Duodenitis | digestive | 11266 | 338422 | 1.02 | 1.01-1.03 | 3.27E-04 |
| 602 | Other disorders of prostate | genitourinary | 2073 | 349822 | 0.96 | 0.94-0.98 | 3.31E-04 |
| 714.1 | Rheumatoid arthritis | musculoskeletal | 7601 | 369947 | 0.98 | 0.97-0.99 | 4.65E-04 |
| 280.1 | Iron deficiency anaemias, unspecified or not due to blood loss | hematopoietic | 17446 | 348201 | 1.01 | 1.01-1.02 | 4.92E-04 |
| 312.3 | Impulse control disorder | mental disorders | 21 | 384036 | 0.67 | 0.53-0.84 | 6.82E-04 |
| 159.2 | Malignant neoplasm of small intestine, including duodenum | neoplasms | 432 | 355250 | 0.92 | 0.88-0.97 | 7.76E-04 |
| 743.11 | Osteoporosis NOS | musculoskeletal | 15202 | 368351 | 1.01 | 1.01-1.02 | 8.58E-04 |
| 709.7 | Unspecified diffuse connective tissue disease | dermatologic | 3772 | 369073 | 1.03 | 1.01-1.04 | 8.96E-04 |
| 415.2 | Chronic pulmonary heart disease | circulatory system | 487 | 369032 | 1.07 | 1.03-1.12 | 9.16E-04 |
| 202.24 | Large cell lymphoma | neoplasms | 1289 | 376925 | 1.04 | 1.02-1.07 | 9.32E-04 |
| 366 | Cataract | sense organs | 40071 | 333751 | 1.01 | 1-1.01 | 1.17E-03 |
| 704 | Diseases of hair and hair follicles | dermatologic | 6062 | 377704 | 0.98 | 0.97-0.99 | 1.24E-03 |
| 261.2 | Vitamin B-complex deficiencies | endocrine/metabolic | 3148 | 374973 | 1.03 | 1.01-1.04 | 1.37E-03 |
| 622.1 | Polyp of corpus uteri | genitourinary | 9219 | 371682 | 0.98 | 0.97-0.99 | 1.42E-03 |
| 285 | Other anaemias | hematopoietic | 23261 | 348201 | 1.01 | 1-1.02 | 1.45E-03 |
| 250.6 | Polyneuropathy in diabetes | endocrine/metabolic | 1053 | 350991 | 1.05 | 1.02-1.08 | 1.54E-03 |
| 443.7 | Peripheral angiopathy in diseases classified elsewhere | circulatory system | 892 | 367618 | 1.05 | 1.02-1.08 | 1.73E-03 |
| 418.1 | Precordial pain | circulatory system | 6944 | 343530 | 1.02 | 1.01-1.03 | 1.81E-03 |
| 962.3 | Hormones and synthetic substitutes causing adverse effects in therapeutic use | injuries & poisonings | 733 | 337400 | 1.06 | 1.02-1.09 | 1.88E-03 |
| 189.12 | Malignant neoplasm of renal pelvis | neoplasms | 194 | 378215 | 0.90 | 0.84-0.96 | 1.96E-03 |
| 496 | Chronic airway obstruction | respiratory | 16653 | 333410 | 1.01 | 1-1.02 | 2.06E-03 |
| 195.1 | Malignant neoplasm, other | neoplasms | 34292 | 324684 | 0.99 | 0.99-1 | 2.38E-03 |
| 780 | Hypothermia/Chills | symptoms | 633 | 385166 | 1.06 | 1.02-1.1 | 2.54E-03 |
| 735.21 | Hammer toe (acquired) | musculoskeletal | 2714 | 364490 | 0.97 | 0.96-0.99 | 2.61E-03 |
| 507 | Pleurisy; pleural effusion | respiratory | 16265 | 354851 | 1.01 | 1-1.02 | 2.65E-03 |
| 728.71 | Contracture of palmar fascia [Dupuytren's disease] | musculoskeletal | 5210 | 339825 | 1.02 | 1.01-1.03 | 2.69E-03 |
| 506 | Empyema and pneumothorax | respiratory | 2352 | 354851 | 1.03 | 1.01-1.05 | 2.78E-03 |
| 496.21 | Obstructive chronic bronchitis | respiratory | 6752 | 333410 | 1.02 | 1.01-1.03 | 2.98E-03 |
| 615 | Endometriosis | genitourinary | 4352 | 375509 | 0.98 | 0.96-0.99 | 3.22E-03 |
| 279 | Disorders involving the immune mechanism | endocrine/metabolic | 103 | 384833 | 1.14 | 1.04-1.24 | 3.35E-03 |
| 204.12 | Lymphoid leukaemia, chronic | neoplasms | 1107 | 376925 | 1.04 | 1.01-1.07 | 4.39E-03 |
| 351 | Other peripheral nerve disorders | neurological | 16181 | 365325 | 1.01 | 1-1.02 | 4.50E-03 |
| 189.2 | Cancer of bladder | neoplasms | 2605 | 378215 | 0.97 | 0.96-0.99 | 4.95E-03 |
| 790.6 | Other abnormal blood chemistry | symptoms | 13876 | 371212 | 0.99 | 0.98-1 | 5.09E-03 |
| 184.2 | Cancer of other female genital organs | neoplasms | 712 | 354792 | 0.95 | 0.92-0.99 | 5.26E-03 |
| 242.3 | Exophthalmos | endocrine/metabolic | 174 | 357107 | 1.10 | 1.03-1.18 | 5.27E-03 |
| 530.9 | Heartburn | digestive | 3226 | 319347 | 0.98 | 0.96-0.99 | 5.29E-03 |
| 371.3 | Inflammation of eyelids | sense organs | 3354 | 370806 | 0.98 | 0.96-0.99 | 5.41E-03 |
| 695.8 | Other specified erythematous conditions | dermatologic | 321 | 373643 | 1.07 | 1.02-1.13 | 5.67E-03 |
| 504 | Other alveolar and parietoalveolar pneumonopathy | respiratory | 1185 | 354851 | 1.04 | 1.01-1.07 | 5.69E-03 |
| 259.2 | Carcinoid syndrome | endocrine/metabolic | 68 | 377675 | 0.85 | 0.75-0.95 | 6.21E-03 |
| 255.21 | Glucocorticoid deficiency | endocrine/metabolic | 782 | 377675 | 1.05 | 1.01-1.08 | 6.39E-03 |
| 348.8 | Encephalopathy, not elsewhere classified | neurological | 337 | 359150 | 1.07 | 1.02-1.13 | 6.39E-03 |
| 870.3 | Other open wound of head and face | injuries & poisonings | 6043 | 371072 | 1.02 | 1-1.03 | 6.53E-03 |
| 601.12 | Chronic prostatitis | genitourinary | 1371 | 349822 | 0.97 | 0.94-0.99 | 7.07E-03 |
| 296 | Mood disorders | mental disorders | 197 | 305591 | 0.91 | 0.85-0.97 | 7.08E-03 |
| 172.2 | Other non-epithelial cancer of skin | neoplasms | 32424 | 347193 | 0.99 | 0.99-1 | 7.11E-03 |
| 287.3 | Thrombocytopenia | hematopoietic | 3220 | 380168 | 1.02 | 1.01-1.04 | 7.26E-03 |
| 250.3 | Insulin pump user | endocrine/metabolic | 112 | 350991 | 1.12 | 1.03-1.22 | 7.59E-03 |
| 338.2 | Chronic pain | neurological | 772 | 384421 | 1.05 | 1.01-1.08 | 7.96E-03 |
| 702.1 | Actinic keratosis | dermatologic | 5778 | 375343 | 0.98 | 0.97-1 | 8.08E-03 |
| 325 | Phlebitis and thrombophlebitis of intracranial venous sinuses | neurological | 89 | 384118 | 1.14 | 1.03-1.25 | 8.11E-03 |
| 53 | Herpes zoster | infectious diseases | 658 | 376556 | 1.05 | 1.01-1.09 | 8.41E-03 |
| 618.2 | Uterine/Uterovaginal prolapse | genitourinary | 6418 | 371651 | 0.98 | 0.97-1 | 8.71E-03 |
| 211 | Benign neoplasm of other parts of digestive system | neoplasms | 11465 | 358216 | 0.99 | 0.98-1 | 8.78E-03 |
| 709.6 | Other specified diffuse diseases of connective tissue | dermatologic | 94 | 369073 | 1.13 | 1.03-1.24 | 8.87E-03 |
| 250.2 | Type 2 diabetes | endocrine/metabolic | 31332 | 350991 | 1.01 | 1-1.01 | 9.04E-03 |
| 695.81 | Erythema nodosum | dermatologic | 105 | 373643 | 1.12 | 1.03-1.23 | 9.19E-03 |
| 528 | Diseases of the oral soft tissues, excluding lesions specific for gingiva and tongue | digestive | 2927 | 377440 | 1.02 | 1.01-1.04 | 9.51E-03 |
| 756.1 | Congenital anomalies of abdominal wall; diaphram | congenital anomalies | 40 | 383865 | 0.81 | 0.69-0.95 | 9.89E-03 |
| 572 | Ascites (non malignant) | digestive | 3366 | 364216 | 1.02 | 1.01-1.04 | 9.92E-03 |
| 578.1 | Hematemesis | digestive | 3162 | 348925 | 0.98 | 0.96-0.99 | 9.96E-03 |
| 365 | Glaucoma | sense organs | 9441 | 358182 | 0.99 | 0.98-1 | 1.00E-02 |
| 282.8 | Other hemoglobinopathies | hematopoietic | 179 | 348201 | 0.91 | 0.84-0.98 | 1.05E-02 |
| 260.1 | Cachexia | endocrine/metabolic | 226 | 374973 | 0.92 | 0.86-0.98 | 1.05E-02 |
| 722.9 | Other and unspecified disc disorder | musculoskeletal | 6896 | 357431 | 1.01 | 1-1.03 | 1.06E-02 |
| 366.3 | Traumatic cataract | sense organs | 124 | 333751 | 1.11 | 1.02-1.2 | 1.06E-02 |
| 366.2 | Senile cataract | sense organs | 27635 | 333751 | 1.01 | 1-1.01 | 1.06E-02 |
| 244.1 | Secondary hypothyroidism | endocrine/metabolic | 2295 | 357107 | 1.03 | 1.01-1.05 | 1.14E-02 |
| 360 | Disorders of the globe | sense organs | 127 | 358182 | 0.90 | 0.82-0.98 | 1.17E-02 |
| 530.11 | GERD | digestive | 33917 | 319347 | 0.99 | 0.99-1 | 1.18E-02 |
| 726.4 | Calcaneal spur; Exostosis NOS | musculoskeletal | 116 | 339825 | 1.11 | 1.02-1.21 | 1.23E-02 |
| 145.3 | Cancer of major salivary glands | neoplasms | 253 | 382236 | 1.08 | 1.02-1.14 | 1.24E-02 |
| 444.1 | Arterial embolism and thrombosis of lower extremity artery | circulatory system | 973 | 367618 | 1.04 | 1.01-1.07 | 1.24E-02 |
| 497 | Bronchitis | respiratory | 1034 | 333410 | 0.96 | 0.94-0.99 | 1.31E-02 |
| 246 | Other disorders of thyroid | endocrine/metabolic | 1084 | 357107 | 1.04 | 1.01-1.07 | 1.39E-02 |
| 374.2 | Lagophthalmos | sense organs | 79 | 370806 | 1.13 | 1.03-1.25 | 1.40E-02 |
| 585.3 | Chronic renal failure [CKD] | genitourinary | 9051 | 353615 | 1.01 | 1-1.02 | 1.49E-02 |
| 279.11 | Deficiency of humoral immunity | endocrine/metabolic | 310 | 384833 | 1.07 | 1.01-1.12 | 1.53E-02 |
| 586.2 | Cyst of kidney, acquired | genitourinary | 4227 | 353615 | 0.98 | 0.97-1 | 1.56E-02 |
| 446.4 | Wegener's granulomatosis | circulatory system | 260 | 367618 | 0.93 | 0.88-0.99 | 1.56E-02 |
| 394.7 | Disease of tricuspid valve | circulatory system | 4570 | 367750 | 1.02 | 1-1.03 | 1.58E-02 |
| 803 | Fracture of upper limb | injuries & poisonings | 721 | 351452 | 0.96 | 0.92-0.99 | 1.63E-02 |
| 709.4 | Polymyositis | dermatologic | 72 | 369073 | 1.14 | 1.02-1.26 | 1.67E-02 |
| 228 | Hemangioma and lymphangioma, any site | neoplasms | 2464 | 383335 | 0.98 | 0.96-1 | 1.70E-02 |
| 346.3 | Nonspecific abnormal findings in cerebrospinal fluid | neurological | 49 | 359150 | 1.17 | 1.03-1.32 | 1.73E-02 |
| 281.11 | Pernicious anemia | hematopoietic | 1278 | 348201 | 1.03 | 1.01-1.06 | 1.77E-02 |
| 165.1 | Cancer of bronchus; lung | neoplasms | 5634 | 379337 | 1.02 | 1-1.03 | 1.78E-02 |
| 561 | Symptoms involving digestive system | digestive | 24033 | 276715 | 1.01 | 1-1.01 | 1.82E-02 |
| 550.1 | Inguinal hernia | digestive | 21881 | 316944 | 0.99 | 0.99-1 | 2.03E-02 |
| 454.1 | Varicose veins of lower extremity | circulatory system | 13141 | 339361 | 0.99 | 0.98-1 | 2.04E-02 |
| 193 | Thyroid cancer | neoplasms | 819 | 382744 | 0.96 | 0.93-0.99 | 2.06E-02 |
| 569.2 | Gastrointestinal complications | digestive | 936 | 349381 | 1.04 | 1.01-1.07 | 2.13E-02 |
| 204.1 | Lymphoid leukemia | neoplasms | 125 | 376925 | 1.10 | 1.01-1.19 | 2.16E-02 |
| 370 | Keratitis | sense organs | 292 | 370806 | 1.06 | 1.01-1.12 | 2.16E-02 |
| 610.8 | Other specified benign mammary dysplasias | genitourinary | 860 | 377949 | 0.96 | 0.93-0.99 | 2.16E-02 |
| 736 | Other acquired deformities of limbs | musculoskeletal | 3194 | 364490 | 1.02 | 1-1.04 | 2.17E-02 |
| 520.1 | Hereditary disturbances in tooth structure | digestive | 61 | 371419 | 1.14 | 1.02-1.28 | 2.22E-02 |
| 596.5 | Functional disorders of bladder | genitourinary | 1886 | 363549 | 0.98 | 0.95-1 | 2.27E-02 |
| 350.6 | Disturbances of sensation of smell and taste | neurological | 122 | 374330 | 1.10 | 1.01-1.19 | 2.34E-02 |
| 389.2 | Conductive hearing loss | sense organs | 643 | 371732 | 1.04 | 1.01-1.08 | 2.38E-02 |
| 260.6 | Anorexia | endocrine/metabolic | 2218 | 374973 | 1.02 | 1-1.04 | 2.38E-02 |
| 379.4 | Anomalies of pupillary function | sense organs | 327 | 366602 | 0.94 | 0.89-0.99 | 2.41E-02 |
| 365.2 | Primary angle-closure glaucoma | sense organs | 1478 | 358182 | 0.97 | 0.95-1 | 2.43E-02 |
| 807 | Fracture of ribs | injuries & poisonings | 3328 | 351452 | 1.02 | 1-1.04 | 2.47E-02 |
| 705.8 | Hyperhidrosis | dermatologic | 1114 | 373939 | 1.03 | 1-1.06 | 2.51E-02 |
| 210 | Benign neoplasm of lip, oral cavity, and pharynx | neoplasms | 1339 | 382236 | 1.03 | 1-1.06 | 2.51E-02 |
| 702.2 | Seborrheic keratosis | dermatologic | 5265 | 375343 | 0.99 | 0.97-1 | 2.53E-02 |
| 446.8 | Thrombotic microangiopathy | circulatory system | 32 | 367618 | 1.19 | 1.02-1.39 | 2.56E-02 |
| 735.2 | Acquired toe deformities | musculoskeletal | 2839 | 364490 | 0.98 | 0.96-1 | 2.58E-02 |
| 619.2 | Disorders of uterus, NEC | genitourinary | 4157 | 374787 | 0.98 | 0.97-1 | 2.59E-02 |
| 529.1 | Glossitis | digestive | 470 | 377440 | 1.05 | 1.01-1.09 | 2.60E-02 |
| 283.1 | Autoimmune hemolytic anemias | hematopoietic | 212 | 348201 | 1.07 | 1.01-1.14 | 2.62E-02 |
| 599.4 | Urinary incontinence | genitourinary | 13008 | 345555 | 0.99 | 0.98-1 | 2.66E-02 |
| 380 | Disorders of external ear | sense organs | 932 | 383607 | 1.03 | 1-1.07 | 2.70E-02 |
| 527.8 | Other specified diseases of the salivary glands | digestive | 183 | 377440 | 1.08 | 1.01-1.15 | 2.72E-02 |
| 603.1 | Hydrocele | genitourinary | 1974 | 352977 | 0.98 | 0.96-1 | 2.72E-02 |
| 131 | Protozoan infection | infectious diseases | 31 | 384318 | 0.82 | 0.68-0.98 | 2.80E-02 |
| 573.2 | Liver replaced by transplant | digestive | 141 | 364216 | 1.09 | 1.01-1.18 | 2.92E-02 |
| 475 | Chronic sinusitis | respiratory | 3703 | 358682 | 0.98 | 0.97-1 | 2.96E-02 |
| 537 | Other disorders of stomach and duodenum | digestive | 6368 | 338422 | 1.01 | 1-1.03 | 3.08E-02 |
| 741.2 | Stiffness of joint | musculoskeletal | 827 | 375807 | 1.04 | 1-1.07 | 3.08E-02 |
| 591 | Urinary tract infection | genitourinary | 23887 | 342565 | 0.99 | 0.99-1 | 3.09E-02 |
| 710.11 | Acute osteomyelitis | musculoskeletal | 69 | 310203 | 1.13 | 1.01-1.25 | 3.10E-02 |
| 588 | Disorders resulting from impaired renal function | genitourinary | 153 | 353615 | 1.08 | 1.01-1.17 | 3.15E-02 |
| 301 | Personality disorders | mental disorders | 414 | 305591 | 0.95 | 0.91-1 | 3.17E-02 |
| 571.81 | Portal hypertension | digestive | 1225 | 364216 | 1.03 | 1-1.06 | 3.22E-02 |
| 359.2 | Myopathy | neurological | 612 | 380976 | 1.04 | 1-1.08 | 3.23E-02 |
| 750.11 | Esophageal atresia/tracheoesophageal fistula | congenital anomalies | 53 | 382353 | 0.86 | 0.75-0.99 | 3.25E-02 |
| 379.2 | Disorders of vitreous body | sense organs | 3319 | 366602 | 1.02 | 1-1.03 | 3.29E-02 |
| 198.1 | Secondary malignancy of lymph nodes | neoplasms | 10408 | 324684 | 0.99 | 0.98-1 | 3.31E-02 |
| 791 | Gangrene | symptoms | 1499 | 384300 | 1.03 | 1-1.05 | 3.32E-02 |
| 425.1 | Primary/intrinsic cardiomyopathies | circulatory system | 2259 | 378560 | 0.98 | 0.96-1 | 3.46E-02 |
| 79.1 | Varicella infection | infectious diseases | 77 | 376556 | 1.12 | 1.01-1.24 | 3.51E-02 |
| 286.1 | Congenital coagulation defects | hematopoietic | 53 | 380168 | 0.87 | 0.76-0.99 | 3.66E-02 |
| 962 | Poisoning by hormones and synthetic substitutes | injuries & poisonings | 250 | 337400 | 1.06 | 1-1.13 | 3.67E-02 |
| 597 | Other disorders of urethra and urinary tract | genitourinary | 1132 | 363549 | 0.97 | 0.94-1 | 3.71E-02 |
| 327.41 | Organic or persistent insomnia | neurological | 544 | 376361 | 1.04 | 1-1.08 | 3.78E-02 |
| 261.41 | Rickets or osteomalacia | endocrine/metabolic | 109 | 374973 | 1.10 | 1.01-1.19 | 3.79E-02 |
| 343 | Infantile cerebral palsy | neurological | 198 | 359150 | 0.93 | 0.87-1 | 3.79E-02 |
| 474.2 | Chronic tonsillitis and adenoiditis | respiratory | 1308 | 358682 | 0.97 | 0.95-1 | 3.82E-02 |
| 277.51 | Lipoprotein disorders | endocrine/metabolic | 98 | 381071 | 1.10 | 1.01-1.21 | 3.86E-02 |
| 531.2 | Gastric ulcer | digestive | 6720 | 374413 | 1.01 | 1-1.02 | 3.91E-02 |
| 687.4 | Disturbance of skin sensation | dermatologic | 4720 | 376346 | 1.01 | 1-1.03 | 3.91E-02 |
| 966 | Poisoning by anticonvulsants and anti-Parkinsonism drugs | injuries & poisonings | 746 | 337400 | 0.96 | 0.93-1 | 3.95E-02 |
| 531.5 | Gastrojejunal ulcer | digestive | 76 | 374413 | 0.89 | 0.8-0.99 | 3.97E-02 |
| 361 | Retinal detachments and defects | sense organs | 2911 | 358182 | 1.02 | 1-1.04 | 4.00E-02 |
| 989 | Toxic effect of other substances, chiefly nonmedicinal as to source | injuries & poisonings | 188 | 385045 | 0.93 | 0.87-1 | 4.08E-02 |
| 750.22 | Congenital anomaly of gallbladder, bile ducts, liver, pancreas | congenital anomalies | 205 | 382353 | 1.07 | 1-1.14 | 4.09E-02 |
| 870.1 | Open wound or laceration of eye or eyelid | injuries & poisonings | 1105 | 371072 | 1.03 | 1-1.06 | 4.14E-02 |
| 556.1 | Ulceration of intestine | digestive | 1323 | 276715 | 0.97 | 0.95-1 | 4.24E-02 |
| 260 | Protein-calorie malnutrition | endocrine/metabolic | 549 | 374973 | 1.04 | 1-1.08 | 4.29E-02 |
| 696.4 | Psoriasis | dermatologic | 3577 | 366117 | 0.98 | 0.97-1 | 4.29E-02 |
| 720 | Spinal stenosis | musculoskeletal | 8355 | 357431 | 1.01 | 1-1.02 | 4.33E-02 |
| 975 | Poisoning by agents primarily acting on the smooth and skeletal muscles and respiratory system | injuries & poisonings | 93 | 337400 | 0.90 | 0.82-1 | 4.35E-02 |
| 189 | Cancer of urinary organs (incl. kidney and bladder) | neoplasms | 5075 | 378215 | 0.99 | 0.97-1 | 4.56E-02 |
| 614.52 | Vaginitis and vulvovaginitis | genitourinary | 526 | 375509 | 0.96 | 0.92-1 | 4.59E-02 |

CI = confidence interval; OR = odds ratio.

# **Supplementary Table 3.** The associations between genetic liability to coeliac disease and 68 clinical outcomes using genetic instruments including and excluding *MHC* SNPs.

|  |  | **All SNPs** | | | **SNPs not in xMHC region** | | |
| --- | --- | --- | --- | --- | --- | --- | --- |
| **Phecode** | **Clinical outcome** | **OR** | **95% CI** | ***Pval*** | **OR** | **95% CI** | ***Pval*** |
| 557.1 | Coeliac disease | 1.62 | 1.60-1.65 | 0.00E+00 | 2.15 | 1.66-2.79 | 9.02E-09 |
| 250.1 | Type 1 diabetes | 1.09 | 1.07-1.11 | 4.54E-27 | 1.01 | 0.80-1.28 | 0.933 |
| 250.13 | Type 1 diabetes with ophthalmic manifestations | 1.16 | 1.13-1.20 | 1.00E-19 | 1.07 | 0.65-1.76 | 0.795 |
| 250.11 | Type 1 diabetes with ketoacidosis | 1.21 | 1.16-1.27 | 1.81E-17 | 1.17 | 0.59-2.33 | 0.654 |
| 242.1 | Graves' disease | 1.13 | 1.10-1.17 | 8.86E-15 | 1.43 | 0.88-2.31 | 0.148 |
| 593 | Hematuria | 0.98 | 0.97-0.98 | 3.61E-12 | 1.02 | 0.92-1.12 | 0.722 |
| 242 | Thyrotoxicosis with or without goiter | 1.06 | 1.04-1.08 | 2.46E-11 | 1.37 | 1.05-1.77 | 0.019 |
| 250.14 | Type 1 diabetes with neurological manifestations | 1.19 | 1.13-1.26 | 2.75E-10 | 0.75 | 0.32-1.74 | 0.504 |
| 709.2 | Sicca syndrome | 1.10 | 1.07-1.13 | 7.45E-10 | 1.37 | 0.88-2.14 | 0.163 |
| 185 | Cancer of prostate | 0.98 | 0.97-0.98 | 4.53E-09 | 0.95 | 0.84-1.08 | 0.460 |
| 251.1 | Hypoglycemia | 1.05 | 1.03-1.07 | 1.05E-08 | 0.91 | 0.70-1.18 | 0.468 |
| 70.4 | Chronic hepatitis | 1.13 | 1.08-1.18 | 2.40E-08 | 1.35 | 0.70-2.60 | 0.374 |
| 695.7 | Prurigo and Lichen | 1.07 | 1.04-1.10 | 5.66E-08 | 0.70 | 0.48-1.01 | 0.056 |
| 250.7 | Diabetic retinopathy | 1.04 | 1.03-1.06 | 2.59E-07 | 1.20 | 0.94-1.53 | 0.145 |
| 580.2 | Nephrotic syndrome without mention of glomerulonephritis | 1.08 | 1.05-1.11 | 4.02E-07 | 0.86 | 0.55-1.33 | 0.492 |
| 735.3 | Hallux valgus (Bunion) | 0.97 | 0.96-0.98 | 4.72E-07 | 0.99 | 0.85-1.16 | 0.907 |
| 695.42 | Systemic lupus erythematosus | 1.10 | 1.06-1.14 | 8.22E-07 | 1.48 | 0.83-2.65 | 0.180 |
| 443.1 | Raynaud's syndrome | 1.05 | 1.03-1.06 | 1.10E-06 | 1.23 | 0.95-1.60 | 0.120 |
| 697 | Sarcoidosis | 1.08 | 1.04-1.11 | 1.23E-06 | 1.50 | 0.96-2.34 | 0.074 |
| 706.2 | Sebaceous cyst | 0.98 | 0.97-0.99 | 2.97E-06 | 0.80 | 0.70-0.92 | 0.002 |
| 250.23 | Type 2 diabetes with ophthalmic manifestations | 1.04 | 1.02-1.05 | 4.90E-06 | 1.16 | 0.92-1.47 | 0.198 |
| 596.1 | Bladder neck obstruction | 0.96 | 0.94-0.98 | 9.87E-06 | 0.78 | 0.59-1.02 | 0.067 |
| 600 | Hyperplasia of prostate | 0.98 | 0.98-0.99 | 1.35E-05 | 1.02 | 0.92-1.13 | 0.709 |
| 180.3 | Cervical intraepithelial neoplasia [CIN] [Cervical dysplasia] | 0.97 | 0.96-0.99 | 1.43E-05 | 1.18 | 0.99-1.41 | 0.069 |
| 715.2 | Ankylosing spondylitis | 0.93 | 0.89-0.96 | 1.55E-05 | 1.29 | 0.79-2.12 | 0.311 |
| 250 | Diabetes mellitus | 1.01 | 1.01-1.02 | 1.66E-05 | 0.95 | 0.87-1.03 | 0.185 |
| 496.3 | Bronchiectasis | 0.97 | 0.96-0.98 | 1.74E-05 | 1.04 | 0.85-1.26 | 0.708 |
| 580.12 | Non-proliferative glomerulonephritis | 1.13 | 1.07-1.20 | 2.66E-05 | 0.81 | 0.33-1.99 | 0.645 |
| 244.4 | Hypothyroidism NOS | 1.01 | 1.01-1.02 | 3.69E-05 | 1.06 | 0.97-1.17 | 0.197 |
| 717 | Polymyalgia Rheumatica | 0.97 | 0.95-0.98 | 4.06E-05 | 0.96 | 0.76-1.20 | 0.696 |
| 555 | Inflammatory bowel disease and other gastroenteritis and colitis | 1.01 | 1.01-1.02 | 4.33E-05 | 1.04 | 0.95-1.14 | 0.381 |
| 594.1 | Calculus of kidney | 0.97 | 0.96-0.99 | 4.82E-05 | 0.99 | 0.82-1.20 | 0.949 |
| 557 | Intestinal malabsorption (non-coeliac) | 1.08 | 1.04-1.12 | 5.54E-05 | 0.79 | 0.45-1.38 | 0.412 |
| 477 | Epistaxis or throat hemorrhage | 0.97 | 0.96-0.98 | 6.42E-05 | 0.87 | 0.70-1.08 | 0.206 |
| 707 | Chronic ulcer of skin | 1.03 | 1.02-1.05 | 7.21E-05 | 1.10 | 0.86-1.39 | 0.454 |
| 558 | Noninfectious gastroenteritis | 1.01 | 1.01-1.02 | 8.86E-05 | 1.03 | 0.93-1.14 | 0.595 |
| 695.41 | Cutaneous lupus erythematosus | 1.12 | 1.05-1.18 | 1.41E-04 | 1.64 | 0.70-3.84 | 0.255 |
| 250.24 | Type 2 diabetes with neurological manifestations | 1.05 | 1.02-1.08 | 1.64E-04 | 1.19 | 0.81-1.74 | 0.372 |
| 870 | Open wounds of head; neck; and trunk | 1.10 | 1.04-1.15 | 2.04E-04 | 0.83 | 0.40-1.72 | 0.615 |
| 300.1 | Anxiety disorder | 0.99 | 0.98-0.99 | 2.16E-04 | 0.97 | 0.87-1.08 | 0.546 |
| 202.2 | Non-Hodgkins lymphoma | 1.03 | 1.01-1.05 | 2.89E-04 | 1.01 | 0.78-1.30 | 0.943 |
| 535.6 | Duodenitis | 1.02 | 1.01-1.03 | 3.27E-04 | 1.04 | 0.91-1.18 | 0.578 |
| 602 | Other disorders of prostate | 0.96 | 0.94-0.98 | 3.31E-04 | 1.13 | 0.83-1.52 | 0.443 |
| 714.1 | Rheumatoid arthritis | 0.98 | 0.97-0.99 | 4.65E-04 | 0.92 | 0.78-1.08 | 0.292 |
| 280.1 | Iron deficiency anemias, unspecified or not due to blood loss | 1.01 | 1.01-1.02 | 4.92E-04 | 1.12 | 1.01-1.25 | 0.033 |
| 159.2 | Malignant neoplasm of small intestine, including duodenum | 0.92 | 0.88-0.97 | 7.76E-04 | 0.80 | 0.41-1.55 | 0.509 |
| 743.11 | Osteoporosis NOS | 1.01 | 1.01-1.02 | 8.58E-04 | 1.12 | 1.00-1.25 | 0.059 |
| 709.7 | Unspecified diffuse connective tissue disease | 1.03 | 1.01-1.04 | 8.96E-04 | 1.20 | 0.96-1.50 | 0.114 |
| 415.2 | Chronic pulmonary heart disease | 1.07 | 1.03-1.12 | 9.16E-04 | 0.92 | 0.50-1.72 | 0.803 |
| 202.24 | Large cell lymphoma | 1.04 | 1.02-1.07 | 9.32E-04 | 1.00 | 0.69-1.47 | 0.982 |
| 366 | Cataract | 1.01 | 1.00-1.01 | 1.17E-03 | 1.00 | 0.93-1.08 | 0.964 |
| 704 | Diseases of hair and hair follicles | 0.98 | 0.97-0.99 | 1.24E-03 | 0.76 | 0.64-0.91 | 0.003 |
| 261.2 | Vitamin B-complex deficiencies | 1.03 | 1.01-1.04 | 1.37E-03 | 1.13 | 0.89-1.44 | 0.321 |
| 622.1 | Polyp of corpus uteri | 0.98 | 0.97-0.99 | 1.42E-03 | 1.03 | 0.89-1.19 | 0.703 |
| 285 | Other anemias | 1.01 | 1.00-1.02 | 1.45E-03 | 1.00 | 0.91-1.10 | 0.948 |
| 250.6 | Polyneuropathy in diabetes | 1.05 | 1.02-1.08 | 1.54E-03 | 1.17 | 0.77-1.77 | 0.476 |
| 443.7 | Peripheral angiopathy in diseases classified elsewhere | 1.05 | 1.02-1.08 | 1.73E-03 | 1.32 | 0.83-2.08 | 0.240 |
| 418.1 | Precordial pain | 1.02 | 1.01-1.03 | 1.81E-03 | 0.95 | 0.80-1.12 | 0.542 |
| 962.3 | Hormones and synthetic substitutes causing adverse effects in therapeutic use | 1.06 | 1.02-1.09 | 1.88E-03 | 0.69 | 0.42-1.15 | 0.159 |
| 496 | Chronic airway obstruction | 1.01 | 1.00-1.02 | 2.06E-03 | 0.97 | 0.87-1.09 | 0.649 |
| 195.1 | Malignant neoplasm, other | 0.99 | 0.99-1.00 | 2.38E-03 | 0.96 | 0.88-1.04 | 0.275 |
| 780 | Hypothermia/Chills | 1.06 | 1.02-1.10 | 2.54E-03 | 0.82 | 0.48-1.42 | 0.486 |
| 735.21 | Hammer toe (acquired) | 0.97 | 0.96-0.99 | 2.61E-03 | 0.82 | 0.63-1.07 | 0.142 |
| 507 | Pleurisy; pleural effusion | 1.01 | 1.00-1.02 | 2.65E-03 | 1.07 | 0.96-1.19 | 0.245 |
| 728.71 | Contracture of palmar fascia [Dupuytren's disease] | 1.02 | 1.01-1.03 | 2.69E-03 | 1.04 | 0.86-1.26 | 0.678 |
| 506 | Empyema and pneumothorax | 1.03 | 1.01-1.05 | 2.78E-03 | 1.01 | 0.76-1.34 | 0.937 |
| 496.21 | Obstructive chronic bronchitis | 1.02 | 1.01-1.03 | 2.98E-03 | 0.89 | 0.75-1.06 | 0.193 |

CI = confidence interval; OR = odds ratio.

# **Supplementary Table 4.** The associations between genetic liability to coeliac disease and 68 clinical outcomes by age stratification

|  |  | **All participants** | | | **Baseline age ≤ 60 years** | | | **Baseline age > 60 years** | | |
| --- | --- | --- | --- | --- | --- | --- | --- | --- | --- | --- |
| **Phecode** | **Clinical outcome** | **OR** | **95% CI** | ***Pval*** | **OR** | **95% CI** | ***Pval*** | **OR** | **95% CI** | ***Pval*** |
| 557.1 | Coeliac disease | 1.62 | 1.60-1.65 | 0.00E+00 | 1.60 | 1.57-1.64 | 0.00E+00 | 1.65 | 1.61-1.69 | 0.00E+00 |
| 250.1 | Type 1 diabetes | 1.09 | 1.07-1.11 | 4.54E-27 | 1.12 | 1.09-1.14 | 1.70E-23 | 1.06 | 1.04-1.09 | 2.68E-07 |
| 250.13 | Type 1 diabetes with ophthalmic manifestations | 1.16 | 1.13-1.20 | 1.00E-19 | 1.19 | 1.14-1.24 | 1.51E-14 | 1.13 | 1.08-1.19 | 4.47E-07 |
| 250.11 | Type 1 diabetes with ketoacidosis | 1.21 | 1.16-1.27 | 1.81E-17 | 1.18 | 1.11-1.25 | 4.67E-08 | 1.27 | 1.18-1.36 | 1.74E-11 |
| 242.1 | Graves' disease | 1.13 | 1.10-1.17 | 8.86E-15 | 1.13 | 1.08-1.17 | 7.06E-09 | 1.15 | 1.09-1.21 | 1.97E-07 |
| 593 | Hematuria | 0.98 | 0.97-0.98 | 3.61E-12 | 0.98 | 0.97-0.99 | 8.07E-04 | 0.97 | 0.96-0.98 | 1.53E-10 |
| 242 | Thyrotoxicosis with or without goiter | 1.06 | 1.04-1.08 | 2.46E-11 | 1.08 | 1.05-1.10 | 1.27E-08 | 1.05 | 1.02-1.07 | 1.62E-04 |
| 250.14 | Type 1 diabetes with neurological manifestations | 1.19 | 1.13-1.26 | 2.75E-10 | 1.20 | 1.12-1.29 | 6.93E-07 | 1.18 | 1.09-1.28 | 8.92E-05 |
| 709.2 | Sicca syndrome | 1.10 | 1.07-1.13 | 7.45E-10 | 1.13 | 1.08-1.18 | 4.32E-08 | 1.07 | 1.03-1.12 | 9.62E-04 |
| 185 | Cancer of prostate | 0.98 | 0.97-0.98 | 4.53E-09 | 0.98 | 0.97-0.99 | 5.99E-03 | 0.97 | 0.96-0.98 | 3.41E-07 |
| 251.1 | Hypoglycemia | 1.05 | 1.03-1.07 | 1.05E-08 | 1.09 | 1.06-1.12 | 5.84E-09 | 1.03 | 1.01-1.05 | 9.10E-03 |
| 70.4 | Chronic hepatitis | 1.13 | 1.08-1.18 | 2.40E-08 | 1.15 | 1.09-1.22 | 2.71E-06 | 1.11 | 1.04-1.18 | 1.53E-03 |
| 695.7 | Prurigo and Lichen | 1.07 | 1.04-1.10 | 5.66E-08 | 1.04 | 1.01-1.08 | 1.69E-02 | 1.10 | 1.06-1.14 | 1.17E-07 |
| 250.7 | Diabetic retinopathy | 1.04 | 1.03-1.06 | 2.59E-07 | 1.07 | 1.04-1.10 | 3.33E-07 | 1.03 | 1.01-1.05 | 1.35E-02 |
| 580.2 | Nephrotic syndrome without mention of glomerulonephritis | 1.08 | 1.05-1.11 | 4.02E-07 | 1.12 | 1.07-1.17 | 2.94E-07 | 1.05 | 1.01-1.09 | 2.62E-02 |
| 735.3 | Hallux valgus (Bunion) | 0.97 | 0.96-0.98 | 4.72E-07 | 0.97 | 0.96-0.99 | 4.24E-04 | 0.97 | 0.96-0.99 | 2.90E-04 |
| 695.42 | Systemic lupus erythematosus | 1.10 | 1.06-1.14 | 8.22E-07 | 1.04 | 0.99-1.10 | 1.11E-01 | 1.17 | 1.11-1.24 | 2.14E-08 |
| 443.1 | Raynaud's syndrome | 1.05 | 1.03-1.06 | 1.10E-06 | 1.04 | 1.02-1.07 | 1.67E-03 | 1.05 | 1.02-1.08 | 1.82E-04 |
| 697 | Sarcoidosis | 1.08 | 1.04-1.11 | 1.23E-06 | 1.08 | 1.04-1.13 | 4.12E-05 | 1.07 | 1.02-1.12 | 7.79E-03 |
| 706.2 | Sebaceous cyst | 0.98 | 0.97-0.99 | 2.97E-06 | 0.97 | 0.96-0.99 | 3.36E-05 | 0.98 | 0.97-1.00 | 1.87E-02 |
| 250.23 | Type 2 diabetes with ophthalmic manifestations | 1.04 | 1.02-1.05 | 4.90E-06 | 1.05 | 1.03-1.08 | 2.57E-05 | 1.03 | 1.00-1.05 | 1.49E-02 |
| 596.1 | Bladder neck obstruction | 0.96 | 0.94-0.98 | 9.87E-06 | 0.97 | 0.94-1.00 | 2.22E-02 | 0.96 | 0.93-0.98 | 1.44E-04 |
| 600 | Hyperplasia of prostate | 0.98 | 0.98-0.99 | 1.35E-05 | 0.99 | 0.98-1.00 | 6.30E-02 | 0.98 | 0.97-0.99 | 1.11E-04 |
| 180.3 | Cervical intraepithelial neoplasia [CIN] [Cervical dysplasia] | 0.97 | 0.96-0.99 | 1.43E-05 | 0.98 | 0.96-0.99 | 8.96E-04 | 0.96 | 0.94-0.99 | 3.34E-03 |
| 715.2 | Ankylosing spondylitis | 0.93 | 0.89-0.96 | 1.55E-05 | 0.92 | 0.88-0.97 | 8.56E-04 | 0.93 | 0.88-0.98 | 5.74E-03 |
| 250 | Diabetes mellitus | 1.01 | 1.01-1.02 | 1.66E-05 | 1.02 | 1.01-1.03 | 3.05E-07 | 1.00 | 1.00-1.01 | 2.69E-01 |
| 496.3 | Bronchiectasis | 0.97 | 0.96-0.98 | 1.74E-05 | 0.97 | 0.95-0.99 | 4.68E-03 | 0.97 | 0.96-0.99 | 1.09E-03 |
| 580.12 | Non-proliferative glomerulonephritis | 1.13 | 1.07-1.20 | 2.66E-05 | 1.16 | 1.06-1.26 | 9.18E-04 | 1.12 | 1.03-1.21 | 7.88E-03 |
| 244.4 | Hypothyroidism NOS | 1.01 | 1.01-1.02 | 3.69E-05 | 1.02 | 1.01-1.03 | 8.01E-07 | 1.00 | 1.00-1.01 | 3.82E-01 |
| 717 | Polymyalgia Rheumatica | 0.97 | 0.95-0.98 | 4.06E-05 | 0.98 | 0.95-1.01 | 2.92E-01 | 0.96 | 0.94-0.98 | 3.81E-05 |
| 555 | Inflammatory bowel disease and other gastroenteritis and colitis | 1.01 | 1.01-1.02 | 4.33E-05 | 1.01 | 1.00-1.02 | 4.85E-03 | 1.01 | 1.00-1.02 | 3.08E-03 |
| 594.1 | Calculus of kidney | 0.97 | 0.96-0.99 | 4.82E-05 | 0.97 | 0.95-0.99 | 9.05E-04 | 0.98 | 0.96-1.00 | 1.64E-02 |
| 557 | Intestinal malabsorption (non-coeliac) | 1.08 | 1.04-1.12 | 5.54E-05 | 1.09 | 1.04-1.14 | 6.48E-04 | 1.07 | 1.01-1.13 | 2.85E-02 |
| 477 | Epistaxis or throat hemorrhage | 0.97 | 0.96-0.98 | 6.42E-05 | 0.99 | 0.97-1.02 | 5.44E-01 | 0.96 | 0.94-0.97 | 2.86E-06 |
| 707 | Chronic ulcer of skin | 1.03 | 1.02-1.05 | 7.21E-05 | 1.03 | 1.00-1.05 | 6.09E-02 | 1.04 | 1.02-1.06 | 3.80E-04 |
| 558 | Noninfectious gastroenteritis | 1.01 | 1.01-1.02 | 8.86E-05 | 1.01 | 1.00-1.02 | 1.67E-02 | 1.02 | 1.01-1.03 | 1.57E-03 |
| 695.41 | Cutaneous lupus erythematosus | 1.12 | 1.05-1.18 | 1.41E-04 | 1.10 | 1.02-1.19 | 1.26E-02 | 1.13 | 1.04-1.23 | 3.62E-03 |
| 250.24 | Type 2 diabetes with neurological manifestations | 1.05 | 1.02-1.08 | 1.64E-04 | 1.06 | 1.02-1.10 | 2.30E-03 | 1.04 | 1.01-1.08 | 2.06E-02 |
| 870 | Open wounds of head; neck; and trunk | 1.10 | 1.04-1.15 | 2.04E-04 | 1.09 | 1.02-1.15 | 6.35E-03 | 1.11 | 1.03-1.21 | 1.05E-02 |
| 300.1 | Anxiety disorder | 0.99 | 0.98-0.99 | 2.16E-04 | 0.99 | 0.98-1.00 | 1.17E-02 | 0.98 | 0.97-1.00 | 5.83E-03 |
| 202.2 | Non-Hodgkins lymphoma | 1.03 | 1.01-1.05 | 2.89E-04 | 1.03 | 1.01-1.06 | 1.68E-02 | 1.03 | 1.01-1.05 | 6.33E-03 |
| 535.6 | Duodenitis | 1.02 | 1.01-1.03 | 3.27E-04 | 1.02 | 1.00-1.03 | 1.92E-02 | 1.02 | 1.00-1.03 | 6.39E-03 |
| 602 | Other disorders of prostate | 0.96 | 0.94-0.98 | 3.31E-04 | 0.96 | 0.93-1.00 | 4.66E-02 | 0.96 | 0.94-0.99 | 4.09E-03 |
| 714.1 | Rheumatoid arthritis | 0.98 | 0.97-0.99 | 4.65E-04 | 0.99 | 0.98-1.01 | 2.70E-01 | 0.97 | 0.96-0.99 | 1.92E-04 |
| 280.1 | Iron deficiency anemias, unspecified or not due to blood loss | 1.01 | 1.01-1.02 | 4.92E-04 | 1.01 | 1.00-1.02 | 3.13E-02 | 1.01 | 1.00-1.02 | 6.07E-03 |
| 159.2 | Malignant neoplasm of small intestine, including duodenum | 0.92 | 0.88-0.97 | 7.76E-04 | 0.91 | 0.85-0.98 | 1.19E-02 | 0.93 | 0.88-0.99 | 2.28E-02 |
| 743.11 | Osteoporosis NOS | 1.01 | 1.01-1.02 | 8.58E-04 | 1.03 | 1.01-1.04 | 7.03E-05 | 1.01 | 1.00-1.02 | 2.49E-01 |
| 709.7 | Unspecified diffuse connective tissue disease | 1.03 | 1.01-1.04 | 8.96E-04 | 1.03 | 1.01-1.05 | 8.66E-03 | 1.02 | 1.00-1.05 | 3.93E-02 |
| 415.2 | Chronic pulmonary heart disease | 1.07 | 1.03-1.12 | 9.16E-04 | 1.10 | 1.02-1.18 | 9.45E-03 | 1.06 | 1.01-1.11 | 2.50E-02 |
| 202.24 | Large cell lymphoma | 1.04 | 1.02-1.07 | 9.32E-04 | 1.03 | 0.99-1.07 | 1.35E-01 | 1.05 | 1.02-1.09 | 2.27E-03 |
| 366 | Cataract | 1.01 | 1.00-1.01 | 1.17E-03 | 1.01 | 1.00-1.02 | 2.04E-02 | 1.01 | 1.00-1.01 | 2.13E-02 |
| 704 | Diseases of hair and hair follicles | 0.98 | 0.97-0.99 | 1.24E-03 | 0.98 | 0.97-1.00 | 2.26E-02 | 0.98 | 0.96-1.00 | 2.05E-02 |
| 261.2 | Vitamin B-complex deficiencies | 1.03 | 1.01-1.04 | 1.37E-03 | 1.02 | 1.00-1.05 | 9.06E-02 | 1.03 | 1.01-1.05 | 5.66E-03 |
| 622.1 | Polyp of corpus uteri | 0.98 | 0.97-0.99 | 1.42E-03 | 0.99 | 0.97-1.00 | 3.13E-02 | 0.98 | 0.96-1.00 | 1.54E-02 |
| 285 | Other anemias | 1.01 | 1.00-1.02 | 1.45E-03 | 1.01 | 1.00-1.02 | 1.45E-01 | 1.01 | 1.00-1.02 | 2.76E-03 |
| 250.6 | Polyneuropathy in diabetes | 1.05 | 1.02-1.08 | 1.54E-03 | 1.07 | 1.02-1.11 | 2.38E-03 | 1.03 | 0.99-1.07 | 1.35E-01 |
| 443.7 | Peripheral angiopathy in diseases classified elsewhere | 1.05 | 1.02-1.08 | 1.73E-03 | 1.06 | 1.01-1.11 | 1.94E-02 | 1.04 | 1.00-1.09 | 3.36E-02 |
| 418.1 | Precordial pain | 1.02 | 1.01-1.03 | 1.81E-03 | 1.01 | 0.99-1.02 | 2.83E-01 | 1.03 | 1.01-1.05 | 6.50E-04 |
| 962.3 | Hormones and synthetic substitutes causing adverse effects in therapeutic use | 1.06 | 1.02-1.09 | 1.88E-03 | 1.07 | 1.02-1.12 | 7.87E-03 | 1.04 | 0.99-1.09 | 8.25E-02 |
| 496 | Chronic airway obstruction | 1.01 | 1.00-1.02 | 2.06E-03 | 1.02 | 1.01-1.03 | 1.54E-03 | 1.01 | 1.00-1.02 | 1.35E-01 |
| 195.1 | Malignant neoplasm, other | 0.99 | 0.99-1.00 | 2.38E-03 | 0.99 | 0.98-1.00 | 8.47E-02 | 0.99 | 0.98-1.00 | 1.17E-02 |
| 780 | Hypothermia/Chills | 1.06 | 1.02-1.10 | 2.54E-03 | 1.05 | 0.99-1.11 | 8.77E-02 | 1.06 | 1.01-1.11 | 1.25E-02 |
| 735.21 | Hammer toe (acquired) | 0.97 | 0.96-0.99 | 2.61E-03 | 0.97 | 0.94-1.00 | 3.14E-02 | 0.97 | 0.95-1.00 | 3.31E-02 |
| 507 | Pleurisy; pleural effusion | 1.01 | 1.00-1.02 | 2.65E-03 | 1.00 | 0.99-1.02 | 5.01E-01 | 1.02 | 1.01-1.03 | 1.19E-03 |
| 728.71 | Contracture of palmar fascia [Dupuytren's disease] | 1.02 | 1.01-1.03 | 2.69E-03 | 1.03 | 1.01-1.05 | 3.53E-03 | 1.01 | 1.00-1.03 | 1.31E-01 |
| 506 | Empyema and pneumothorax | 1.03 | 1.01-1.05 | 2.78E-03 | 1.02 | 0.99-1.05 | 3.03E-01 | 1.04 | 1.01-1.07 | 2.06E-03 |
| 496.21 | Obstructive chronic bronchitis | 1.02 | 1.01-1.03 | 2.98E-03 | 1.02 | 1.00-1.04 | 2.97E-02 | 1.02 | 1.00-1.03 | 3.35E-02 |

CI = confidence interval; OR = odds ratio.

# **Supplementary Table 5.** Data sources for 57 outcomes in the FinnGen study R9

| **Clinical outcome** | **Cases** | **Controls** | **Total** |
| --- | --- | --- | --- |
| Coeliac disease | 3690 | 361055 | 364745 |
| Type 1 diabetes with renal complications | 1579 | 308280 | 309859 |
| Type 1 diabetes with ketoacidosis | 2102 | 308280 | 310382 |
| Type 1 diabetes with neurological complications | 1077 | 308280 | 309357 |
| Type 1 diabetes with ophthalmic complications | 5202 | 308280 | 313482 |
| Glomerulonephritis | 1605 | 375672 | 377277 |
| Graves disease, strict definition | 2836 | 374441 | 377277 |
| Chronic hepatitis, not elsewhere classified | 885 | 366450 | 367335 |
| Lupus erythematosus | 652 | 353088 | 353740 |
| Sicca syndrome [Sjögren] | 2495 | 365533 | 368028 |
| Type 1 diabetes, wide definition | 8967 | 308373 | 317340 |
| Intestinal malabsorption | 1228 | 361055 | 362283 |
| Nephrotic syndrome | 853 | 371635 | 372488 |
| Sarcoidosis | 4041 | 371255 | 375296 |
| Lichen simplex chronicus and prurigo | 1703 | 336589 | 338292 |
| Thyrotoxicosis | 8173 | 367407 | 375580 |
| Diabetic hypoglycemia | 7332 | 271817 | 279149 |
| Type 2 diabetes with neurological complications | 1894 | 308280 | 310174 |
| Peripheral angiopathy | 2514 | 271817 | 274331 |
| Diabetic polyneuropathy | 1048 | 374434 | 375482 |
| Diffuse large B-cell lymphoma (controls excluding all cancers) | 1010 | 287137 | 288147 |
| Diabetic retinopathy | 6818 | 344569 | 351387 |
| Type 2 diabetes with ophthalmic complications | 4172 | 308280 | 312452 |
| Chronic ulcer of skin, not elsewhere classified | 1840 | 353088 | 354928 |
| Non-Hodgkin lymphoma, all (controls excluding all cancers) | 928 | 287137 | 288065 |
| Pneumothorax | 1999 | 361836 | 363835 |
| Vitamin B12 deficiency anaemia | 3351 | 360528 | 363879 |
| Mixed connective tissue disease | 1849 | 375428 | 377277 |
| Palmar fascial fibromatosis [Dupuytren] | 5128 | 275212 | 280340 |
| Angina pectoris | 34456 | 313400 | 347856 |
| Other gastritis (incl. Duodenitis) | 7514 | 320387 | 327901 |
| Other noninfective gastroenteritis and colitis | 7988 | 359927 | 367915 |
| Hypothyroidism, strict autoimmune | 40926 | 274069 | 314995 |
| Osteoporosis | 7300 | 358014 | 365314 |
| Inflammatory bowel disease, strict (require KELA) | 7625 | 369652 | 377277 |
| Iron deficiency anaemia | 13689 | 360528 | 374217 |
| COPD | 18266 | 311286 | 329552 |
| Pleural effusion | 4513 | 361836 | 366349 |
| Anaemias | 27371 | 88536 | 115907 |
| Senile cataract | 59522 | 312864 | 372386 |
| Malignant neoplasm (controls excluding all cancers) | 77325 | 287137 | 364462 |
| Anxiety disorders (more control exclusions) | 40191 | 277526 | 317717 |
| Hyperplasia of prostate | 30066 | 119297 | 149363 |
| Uterine polyps | 2871 | 107564 | 110435 |
| Rheumatoid arthritis | 12555 | 240862 | 253417 |
| Follicular cysts of skin and subcutaneous tissue | 5459 | 361140 | 366599 |
| Malignant neoplasm of prostate (controls excluding all cancers) | 13216 | 119948 | 133164 |
| All dysplastic lesions of the cervix uteri | 8025 | 202845 | 210870 |
| Hallux valgus (acquired) | 14726 | 240862 | 255588 |
| Calculus of kidney and ureter | 9713 | 366693 | 376406 |
| Other hammer toe(s) (acquired) | 5625 | 240862 | 246487 |
| Bronchiectasis | 2188 | 311286 | 313474 |
| Polymyalgia rheumatica | 3501 | 365533 | 369034 |
| Other disorders of prostate | 991 | 119297 | 120288 |
| Ankylosing spondylitis | 2860 | 270964 | 273824 |
| Malignant neoplasm of small intestine (controls excluding all cancers) | 495 | 287137 | 287632 |
| Malignant neoplasm of renal pelvis (controls excluding all cancers) | 125 | 287137 | 287262 |

# **Supplementary Table 6.** The associations between genetic liability to coeliac disease and 57 outcomes in the primary and sensitivity Mendelian randomization analyses in FinnGen

| **Outcome** | **IVW-random effects** | | | **Weighted median** | | | **MR-Egger** | | | **Cochrans Q** | **P_Egger intercept** | **MR-PRESSO (outlier removed)** | | |
| --- | --- | --- | --- | --- | --- | --- | --- | --- | --- | --- | --- | --- | --- | --- |
|  | **Beta** | **SE** | ***Pval*** | **Beta** | **SE** | ***Pval*** | **Beta** | **SE** | ***Pval*** |  |  | **Beta** | **SE** | ***Pval*** |
| Coeliac disease | 0.79 | 0.04 | 2.02E-103 | 0.66 | 0.03 | 5.34E-99 | 0.83 | 0.06 | 6.10E-21 | 904 | 0.419 | 0.80 | 0.03 | 2.76E-33 |
| Intestinal malabsorption | 0.21 | 0.02 | 2.05E-22 | 0.20 | 0.03 | 7.66E-12 | 0.22 | 0.03 | 2.20E-08 | 103 | 0.797 | 0.21 | 0.02 | 7.89E-15 |
| Graves disease, strict definition | 0.18 | 0.02 | 1.29E-14 | 0.18 | 0.02 | 9.42E-18 | 0.20 | 0.03 | 3.34E-07 | 252 | 0.331 | 0.18 | 0.02 | 4.01E-13 |
| Thyrotoxicosis | 0.11 | 0.02 | 9.69E-13 | 0.08 | 0.01 | 8.31E-11 | 0.12 | 0.02 | 3.90E-06 | 304 | 0.533 | 0.11 | 0.01 | 5.51E-11 |
| Sicca syndrome [Sjögren] | 0.16 | 0.02 | 9.15E-11 | 0.10 | 0.02 | 6.26E-06 | 0.18 | 0.04 | 1.74E-05 | 258 | 0.529 | 0.12 | 0.02 | 1.55E-08 |
| Lupus erythematosus | 0.21 | 0.04 | 1.83E-08 | 0.16 | 0.04 | 1.32E-04 | 0.22 | 0.06 | 3.66E-04 | 156 | 0.854 | 0.16 | 0.03 | 1.56E-05 |
| Mixed connective tissue disease | 0.10 | 0.02 | 4.05E-08 | 0.04 | 0.02 | 9.56E-02 | 0.08 | 0.03 | 6.02E-03 | 96 | 0.337 | 0.09 | 0.02 | 4.74E-06 |
| Chronic hepatitis, not elsewhere classified | 0.14 | 0.03 | 1.27E-06 | 0.13 | 0.03 | 2.53E-04 | 0.14 | 0.04 | 2.03E-03 | 123 | 0.901 | 0.15 | 0.03 | 1.47E-06 |
| Sarcoidosis | 0.14 | 0.03 | 1.53E-06 | 0.13 | 0.02 | 4.59E-10 | 0.15 | 0.05 | 2.11E-03 | 585 | 0.888 | 0.15 | 0.02 | 8.11E-09 |
| Other noninfective gastroenteritis and colitis | 0.05 | 0.01 | 7.29E-06 | 0.04 | 0.01 | 2.90E-04 | 0.01 | 0.02 | 4.54E-01 | 170 | **0.002** | 0.06 | 0.01 | 1.62E-06 |
| Diabetic polyneuropathy | 0.10 | 0.02 | 1.04E-05 | 0.10 | 0.03 | 2.52E-03 | 0.08 | 0.03 | 1.92E-02 | 91 | 0.550 | 0.10 | 0.02 | 8.14E-05 |
| Diabetic hypoglycemia | 0.08 | 0.02 | 1.30E-04 | 0.05 | 0.01 | 5.71E-04 | 0.09 | 0.03 | 7.17E-03 | 449 | 0.686 | 0.05 | 0.01 | 3.60E-04 |
| Type 1 diabetes with neurological complications | 0.19 | 0.05 | 1.33E-04 | 0.12 | 0.04 | 5.90E-04 | 0.21 | 0.08 | 8.16E-03 | 448 | 0.730 | 0.20 | 0.03 | 5.18E-07 |
| Nephrotic syndrome | 0.08 | 0.02 | 4.03E-04 | 0.09 | 0.03 | 4.56E-03 | 0.05 | 0.03 | 1.64E-01 | 73 | 0.232 |  |  |  |
| Osteoporosis | 0.03 | 0.01 | 5.82E-04 | 0.02 | 0.01 | 1.59E-01 | 0.00 | 0.01 | 9.47E-01 | 87 | **0.003** |  |  |  |
| Diabetic retinopathy | 0.11 | 0.03 | 7.74E-04 | 0.07 | 0.02 | 4.38E-06 | 0.11 | 0.05 | 2.84E-02 | 1214 | 0.925 | 0.07 | 0.01 | 1.46E-05 |
| Palmar fascial fibromatosis [Dupuytren] | 0.03 | 0.01 | 1.02E-03 | 0.04 | 0.01 | 3.45E-04 | 0.05 | 0.01 | 7.53E-04 | 61 | 0.075 |  |  |  |
| Type 1 diabetes with ophthalmic complications | 0.17 | 0.05 | 1.05E-03 | 0.10 | 0.02 | 1.04E-08 | 0.18 | 0.08 | 3.22E-02 | 2407 | 0.922 | 0.11 | 0.03 | 5.77E-04 |
| Type 1 diabetes, wide definition | 0.15 | 0.05 | 1.23E-03 | 0.08 | 0.01 | 9.08E-09 | 0.15 | 0.07 | 3.64E-02 | 3087 | 0.950 | 0.10 | 0.02 | 3.88E-05 |
| Type 1 diabetes with renal complications | 0.15 | 0.05 | 1.34E-03 | 0.06 | 0.03 | 2.03E-02 | 0.13 | 0.07 | 9.80E-02 | 631 | 0.599 | 0.12 | 0.03 | 7.83E-04 |
| COPD | 0.02 | 0.01 | 1.35E-03 | 0.02 | 0.01 | 3.81E-02 | 0.02 | 0.01 | 1.13E-01 | 99 | 0.534 | 0.02 | 0.01 | 2.05E-03 |
| Senile cataract | 0.01 | 0.00 | 1.71E-03 | 0.01 | 0.01 | 1.29E-02 | 0.01 | 0.01 | 3.17E-02 | 108 | 0.823 | 0.02 | 0.00 | 1.09E-06 |
| Non-Hodgkin lymphoma, all (controls excluding all cancers) | 0.06 | 0.02 | 2.03E-03 | 0.05 | 0.03 | 6.89E-02 | 0.04 | 0.03 | 1.49E-01 | 50 | 0.576 |  |  |  |
| Type 2 diabetes with ophthalmic complications | 0.06 | 0.02 | 2.30E-03 | 0.01 | 0.02 | 4.74E-01 | 0.05 | 0.03 | 7.28E-02 | 240 | 0.845 | 0.06 | 0.01 | 3.68E-04 |
| Hyperplasia of prostate | -0.02 | 0.01 | 2.64E-03 | -0.02 | 0.01 | 9.60E-03 | -0.02 | 0.01 | 5.53E-03 | 102 | 0.233 | -0.01 | 0.00 | 1.43E-02 |
| Lichen simplex chronicus and prurigo | 0.06 | 0.02 | 2.79E-03 | 0.00 | 0.02 | 9.33E-01 | 0.02 | 0.03 | 4.87E-01 | 115 | 0.094 | 0.06 | 0.02 | 3.89E-03 |
| Type 1 diabetes with ketoacidosis | 0.17 | 0.06 | 3.44E-03 | 0.11 | 0.03 | 3.84E-05 | 0.18 | 0.09 | 4.41E-02 | 1141 | 0.830 | 0.11 | 0.03 | 8.51E-05 |
| Anaemias | 0.02 | 0.01 | 3.81E-03 | 0.03 | 0.01 | 3.69E-05 | 0.00 | 0.01 | 7.82E-01 | 137 | **0.031** | 0.04 | 0.01 | 1.58E-06 |
| Malignant neoplasm of prostate (controls excluding all cancers) | -0.02 | 0.01 | 4.20E-03 | -0.01 | 0.01 | 1.00E-01 | -0.02 | 0.01 | 1.52E-01 | 80 | 0.592 | -0.02 | 0.01 | 8.99E-03 |
| Vitamin B12 deficiency anaemia | 0.05 | 0.02 | 1.04E-02 | 0.04 | 0.02 | 1.75E-02 | 0.03 | 0.03 | 3.09E-01 | 232 | 0.398 | 0.05 | 0.01 | 9.88E-04 |
| Pleural effusion | 0.02 | 0.01 | 1.53E-02 | 0.02 | 0.01 | 1.22E-01 | 0.01 | 0.01 | 3.11E-01 | 60 | 0.465 |  |  |  |
| Type 2 diabetes with neurological complications | 0.04 | 0.02 | 1.68E-02 | 0.04 | 0.02 | 6.25E-02 | 0.02 | 0.03 | 3.62E-01 | 94 | 0.401 | 0.05 | 0.02 | 5.39E-03 |
| Iron deficiency anaemia | 0.02 | 0.01 | 1.88E-02 | 0.02 | 0.01 | 5.90E-03 | 0.01 | 0.01 | 3.62E-01 | 102 | 0.422 | 0.02 | 0.01 | 1.48E-03 |
| Bronchiectasis | -0.03 | 0.01 | 2.33E-02 | -0.03 | 0.02 | 1.75E-01 | -0.07 | 0.02 | 1.27E-03 | 58 | **0.015** |  |  |  |
| Hypothyroidism, strict autoimmune | 0.03 | 0.01 | 2.51E-02 | 0.04 | 0.01 | 4.26E-06 | 0.01 | 0.02 | 7.36E-01 | 1137 | 0.137 | 0.04 | 0.01 | 6.08E-05 |
| Malignant neoplasm (controls excluding all cancers) | -0.01 | 0.00 | 3.00E-02 | -0.01 | 0.00 | 3.53E-03 | -0.01 | 0.01 | 3.37E-01 | 105 | 0.562 |  |  |  |
| Peripheral angiopathy | 0.04 | 0.02 | 4.80E-02 | 0.05 | 0.02 | 6.09E-02 | 0.04 | 0.03 | 1.71E-01 | 159 | 0.889 | 0.03 | 0.02 | 9.23E-02 |
| Diffuse large B-cell lymphoma (controls excluding all cancers) | 0.05 | 0.03 | 4.91E-02 | 0.04 | 0.03 | 2.29E-01 | 0.06 | 0.04 | 1.68E-01 | 111 | 0.875 | 0.07 | 0.03 | 6.25E-03 |
| Chronic ulcer of skin, not elsewhere classified | 0.03 | 0.02 | 6.24E-02 | 0.03 | 0.02 | 1.65E-01 | 0.01 | 0.02 | 6.16E-01 | 71 | 0.350 |  |  |  |
| Other gastritis (incl. Duodenitis) | 0.01 | 0.01 | 7.22E-02 | 0.01 | 0.01 | 3.38E-01 | 0.00 | 0.01 | 6.67E-01 | 63 | **0.034** |  |  |  |
| Calculus of kidney and ureter | -0.01 | 0.01 | 1.50E-01 | -0.01 | 0.01 | 1.58E-01 | -0.02 | 0.01 | 6.53E-02 | 66 | 0.222 |  |  |  |
| Angina pectoris | 0.01 | 0.01 | 1.72E-01 | 0.01 | 0.01 | 9.90E-02 | 0.01 | 0.01 | 2.76E-01 | 114 | 0.777 | 0.01 | 0.00 | 2.18E-01 |
| Hallux valgus (acquired) | -0.01 | 0.01 | 1.81E-01 | -0.02 | 0.01 | 1.37E-01 | -0.02 | 0.01 | 2.06E-01 | 170 | 0.589 | -0.02 | 0.01 | 7.80E-02 |
| Glomerulonephritis | 0.02 | 0.02 | 1.99E-01 | 0.00 | 0.02 | 8.71E-01 | 0.00 | 0.03 | 8.49E-01 | 74 | 0.176 |  |  |  |
| Other hammer toe(s) (acquired) | -0.01 | 0.01 | 2.55E-01 | -0.04 | 0.02 | 2.06E-02 | -0.02 | 0.02 | 4.26E-01 | 129 | 0.931 | -0.04 | 0.01 | 1.55E-03 |
| Follicular cysts of skin and subcutaneous tissue | 0.01 | 0.01 | 3.27E-01 | 0.00 | 0.01 | 8.85E-01 | 0.01 | 0.01 | 5.88E-01 | 74 | 0.906 |  |  |  |
| Uterine polyps | -0.01 | 0.01 | 3.28E-01 | 0.00 | 0.02 | 8.49E-01 | -0.01 | 0.02 | 5.31E-01 | 50 | 0.966 |  |  |  |
| Polymyalgia rheumatica | 0.02 | 0.02 | 3.66E-01 | -0.02 | 0.02 | 3.53E-01 | 0.00 | 0.04 | 9.58E-01 | 342 | 0.399 | 0.05 | 0.02 | 1.12E-02 |
| All dysplastic lesions of the cervix uteri | 0.01 | 0.02 | 3.71E-01 | 0.03 | 0.01 | 3.68E-02 | 0.04 | 0.02 | 8.34E-02 | 315 | 0.127 | 0.01 | 0.01 | 5.74E-01 |
| Malignant neoplasm of small intestine (controls excluding all cancers) | 0.02 | 0.03 | 4.66E-01 | -0.01 | 0.04 | 7.40E-01 | 0.01 | 0.05 | 9.00E-01 | 79 | 0.648 |  |  |  |
| Pneumothorax | 0.01 | 0.01 | 5.23E-01 | 0.01 | 0.02 | 7.51E-01 | 0.00 | 0.02 | 9.64E-01 | 58 | 0.545 |  |  |  |
| Other disorders of prostate | -0.01 | 0.02 | 5.41E-01 | 0.01 | 0.03 | 7.05E-01 | -0.01 | 0.03 | 6.66E-01 | 63 | 0.961 |  |  |  |
| Anxiety disorders (more control exclusions) | 0.00 | 0.00 | 6.19E-01 | 0.01 | 0.01 | 1.26E-01 | 0.00 | 0.01 | 7.36E-01 | 109 | 0.983 | 0.00 | 0.00 | 9.60E-01 |
| Inflammatory bowel disease, strict (require KELA) | 0.01 | 0.02 | 6.32E-01 | -0.02 | 0.01 | 1.13E-01 | -0.05 | 0.02 | 3.95E-02 | 372 | **0.002** | 0.00 | 0.01 | 8.92E-01 |
| Ankylosing spondylitis | -0.02 | 0.05 | 6.70E-01 | -0.09 | 0.03 | 2.35E-03 | -0.11 | 0.08 | 2.03E-01 | 1384 | 0.188 | 0.02 | 0.03 | 6.12E-01 |
| Malignant neoplasm of renal pelvis (controls excluding all cancers) | -0.01 | 0.06 | 8.75E-01 | -0.01 | 0.08 | 9.32E-01 | -0.02 | 0.10 | 8.32E-01 | 82 | 0.883 |  |  |  |
| Rheumatoid arthritis | 0.00 | 0.03 | 8.82E-01 | -0.07 | 0.01 | 1.39E-10 | -0.06 | 0.04 | 1.82E-01 | 1579 | 0.103 | 0.05 | 0.01 | 1.14E-03 |

# **Supplementary Table 7.** Genetically predicted abundance of *genus Bifidobacterium* in relation to CeD-associated outcomes

| **Outcome** | **OR** | **95% CI** | ***P* val** | **FDR** |
| --- | --- | --- | --- | --- |
| Osteoporosis NOS | 1.56 | 1.21-2.00 | 0.001 | 0.019 |
| Cutaneous lupus erythematosus | 0.29 | 0.13-0.66 | 0.003 | 0.061 |
| Cataract | 1.15 | 1.03-1.29 | 0.013 | 0.165 |
| Graves' disease | 1.59 | 1.07-2.37 | 0.021 | 0.183 |
| Prurigo and Lichen | 1.78 | 1.08-2.95 | 0.024 | 0.183 |
| Hypothyroidism NOS | 0.90 | 0.80-1.01 | 0.068 | 0.431 |
| Celiac disease | 0.74 | 0.52-1.05 | 0.095 | 0.515 |
| Diabetic retinopathy | 1.21 | 0.94-1.57 | 0.143 | 0.545 |
| Unspecified diffuse connective tissue disease | 0.68 | 0.42-1.11 | 0.122 | 0.545 |
| Thyrotoxicosis with or without goiter | 1.19 | 0.94-1.51 | 0.142 | 0.545 |
| Iron deficiency anemias, unspecified or not due to blood loss | 0.88 | 0.73-1.06 | 0.176 | 0.556 |
| Bronchiectasis | 1.37 | 0.87-2.15 | 0.168 | 0.556 |
| Hyperplasia of prostate | 0.90 | 0.78-1.05 | 0.195 | 0.569 |
| Type 2 diabetes with ophthalmic manifestations | 1.24 | 0.89-1.72 | 0.213 | 0.579 |
| Nephrotic syndrome without mention of glomerulonephritis | 0.65 | 0.32-1.33 | 0.236 | 0.596 |
| Type 1 diabetes | 1.14 | 0.91-1.44 | 0.251 | 0.596 |
| Polyneuropathy in diabetes | 0.70 | 0.36-1.34 | 0.279 | 0.623 |
| Non-Hodgkins lymphoma | 0.71 | 0.35-1.41 | 0.328 | 0.637 |
| Type 1 diabetes with neurological manifestations | 0.72 | 0.38-1.38 | 0.326 | 0.637 |
| Type 1 diabetes with ophthalmic manifestations | 1.15 | 0.85-1.55 | 0.369 | 0.637 |
| Chronic hepatitis | 1.38 | 0.68-2.80 | 0.366 | 0.637 |
| Contracture of palmar fascia [Dupuytren's disease] | 1.15 | 0.85-1.55 | 0.368 | 0.637 |
| Hypoglycemia | 1.12 | 0.86-1.44 | 0.400 | 0.644 |
| Peripheral angiopathy in diseases classified elsewhere | 1.18 | 0.77-1.83 | 0.448 | 0.644 |
| Type 1 diabetes with ketoacidosis | 1.20 | 0.75-1.91 | 0.441 | 0.644 |
| Type 2 diabetes with neurological manifestations | 0.83 | 0.51-1.36 | 0.458 | 0.644 |
| Pleurisy; pleural effusion | 0.88 | 0.64-1.20 | 0.413 | 0.644 |
| Malignant neoplasm, other | 1.03 | 0.94-1.13 | 0.556 | 0.755 |
| Large cell lymphoma | 0.84 | 0.43-1.62 | 0.598 | 0.770 |
| Sicca syndrome | 0.90 | 0.59-1.36 | 0.608 | 0.770 |
| Cancer of prostate | 1.04 | 0.84-1.28 | 0.713 | 0.825 |
| Vitamin B-complex deficiencies | 1.07 | 0.74-1.54 | 0.727 | 0.825 |
| Other anemias | 0.97 | 0.81-1.15 | 0.722 | 0.825 |
| Intestinal malabsorption (non-celiac) | 1.11 | 0.61-2.01 | 0.738 | 0.825 |
| Type 1 diabetes with renal manifestations | 1.08 | 0.64-1.83 | 0.777 | 0.844 |
| Chronic airway obstruction | 1.02 | 0.86-1.20 | 0.832 | 0.879 |
| Noninfectious gastroenteritis | 0.99 | 0.78-1.26 | 0.955 | 0.981 |
| Sarcoidosis | 1.00 | 0.72-1.39 | 0.994 | 0.994 |

CeD, celiac disease; CI, confidence interval; FDR, false discovery rate; OR, odds ratio.

# **Supplementary Table 8.** Univariable (UVMR) and multivariable MR (MVMR) analysis on the associations of genetic liability to CeD with osteoporosis and cutaneous lupus erythematosus

|  | **UVMR** |  |  | **MVMR** |  |  |
| --- | --- | --- | --- | --- | --- | --- |
| **Outcome** | **Beta** | **SE** | **P** | **Beta** | **SE** | **P** |
| Osteoporosis NOS | 0.030 | 0.009 | 0.001 | 0.031 | 0.009 | 0.001 |
| Cutaneous lupus erythematosus | 0.208 | 0.037 | 1.83E-08 | 0.208 | 0.04 | 1.45E-07 |

MVMR was adjusted for predicted abundance of *genus Bifidobacterium.*


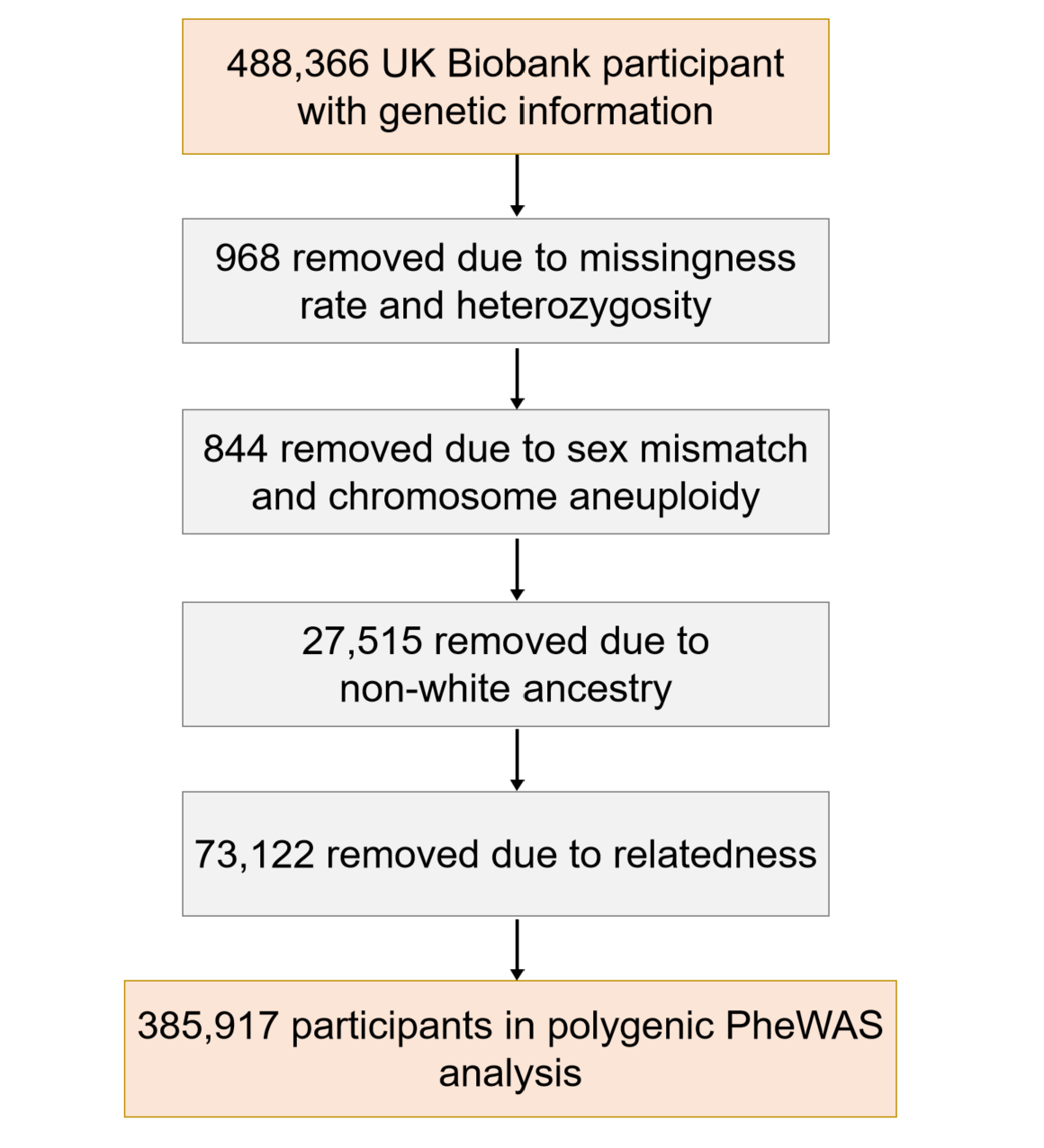


# **Supplementary Figure 1.** Flow chart of study population selection and sample quality control in the UK Biobank.
